# Plant Cell Wall-Like Soft Materials: Micro- and Nanoengineering, Properties, and Applications

**DOI:** 10.1007/s40820-024-01569-0

**Published:** 2025-01-08

**Authors:** Roya Koshani, Mica L. Pitcher, Jingyi Yu, Christine L. Mahajan, Seong H. Kim, Amir Sheikhi

**Affiliations:** 1 Department of Chemical Engineering, The Pennsylvania State University, University Park, PA 16802 USA; 2 Department of Chemistry, The Pennsylvania State University, University Park, PA 16802 USA; 3 Department of Biology, The Pennsylvania State University, University Park, PA 16802 USA; 4 Materials Research Institute, The Pennsylvania State University, University Park, PA 16802 USA; 5 Department of Biomedical Engineering, The Pennsylvania State University, University Park, PA 16802 USA; 6 Huck Institutes of the Life Sciences, The Pennsylvania State University, University Park, PA 16802 USA; 7 Department of Neurosurgery, College of Medicine, The Pennsylvania State University, Hershey, PA 17033 USA

**Keywords:** Synthetic plants, Biomimicry, Acellular wall, Composites, Living materials, Soft matter

## Abstract

Plant cell wall (CW)-like soft materials, referred to as artificial CWs, are composites of assembled polymers containing micro-/nanoparticles or fibers/fibrils that are designed to mimic the composition, structure, and mechanics of plant CWs. CW-like materials have recently emerged to test hypotheses pertaining to the intricate structure–property relationships of native plant CWs or to fabricate functional materials. Here, research on plant CWs and CW-like materials is reviewed by distilling key studies on biomimetic composites primarily composed of plant polysaccharides, including cellulose, pectin, and hemicellulose, as well as organic polymers like lignin. Micro- and nanofabrication of plant CW-like composites, characterization techniques, and in silico studies are reviewed, with a brief overview of current and potential applications. Micro-/nanofabrication approaches include bacterial growth and impregnation, layer-by-layer assembly, film casting, 3-dimensional templating microcapsules, and particle coating. Various characterization techniques are necessary for the comprehensive mechanical, chemical, morphological, and structural analyses of plant CWs and CW-like materials. CW-like materials demonstrate versatility in real-life applications, including biomass conversion, pulp and paper, food science, construction, catalysis, and reaction engineering. This review seeks to facilitate the rational design and thorough characterization of plant CW-mimetic materials, with the goal of advancing the development of innovative soft materials and elucidating the complex structure–property relationships inherent in native CWs.

## Highlights

- This review provides a detailed account of engineered plant cell wall (CW)-mimetic soft materials, which are designed to replicate the intricate composition, structure, and mechanical properties of natural plant CWs.
- Experimental methods to create CW-like materials are reviewed, and relevant characterization techniques, including mechanical, chemical, structural, and morphological analyses, are discussed.
-  The applications of CW-like materials in several fields, including food packaging, edible films, drug delivery, construction materials, and biocatalysis are highlighted.

## Graphical abstract

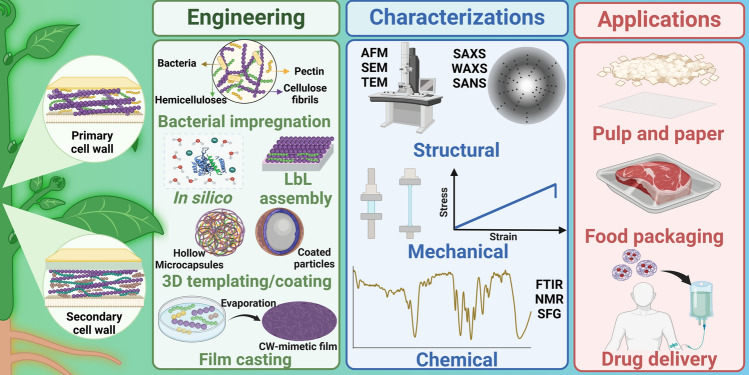

## Introduction

Plant cell walls (CWs) have garnered significant interest as a result of their composition, hierarchical structure, and unique mechanical properties, inspiring the engineering of biomimetic materials. Plant CWs may have high strength, stiffness, and extensibility, depending on growth stages and tissue types [1–3]. To closely mimic the characteristic properties of CWs and construct artificial plant CWs, the contribution of each component as well as the arrangements and interactions of CW building blocks need to be uncovered. To this end, considerable effort has been devoted to investigating the biological mechanisms underlying CW formation [4–6]; however, the isolation of plant CW components for in-depth studies of their native state is non-trivial [7]. The selective removal of CW building blocks via chemical or mechanical treatments may cause degradation and compromise corresponding interactions [8, 9]. To overcome the challenges associated with top-down CW investigations, bottom-up approaches such as developing artificial CWs have emerged [10–13].

CW-like materials generally comprise one to three biopolymer components produced by plants. These materials are classified into either two-dimensional (2D) or three-dimensional (3D) platforms. Fibers, monolithic composites, and films made up of polymers, particles, or their combination are considered as 2D constructs, and 3D models are fabricated using beads, droplets, or plasma membrane templates. The 3D constructs include microcapsules, microspheres, and coated beads [14]. Challenges and opportunities persist in building synthetic CW-like materials. Constructing the cross-lamellate structure and the integration of wall polymers such as lignin remains a key challenge. Additionally, the biomimetic assembly of biopolymers to develop 3D CW-mimetic composites is currently unexplored. Advancements in understanding the structure–property-function relationships of CW combined with advanced material fabrication and synthesis approaches may hold promise to overcome these challenges. Simulation methods, such as finite element analysis and coarse-grained modeling, enable the prediction of assembled CW-like material properties, such as mechanical characteristics, thermodynamic interactions, and transport phenomena [15–17]. Structural characterizations help uncover the effects of the physiochemical properties of CW polymers and inter-fibril interactions on material properties [15]. Additionally, advanced approaches such as bacteria-enabled in situ and ex situ material syntheses, microfluidics, and additive manufacturing may offer solutions for creating more complex, functional, and scalable CW-like materials.

Current studies have concentrated on fabricating plant CW-like materials either to elucidate CW structures or for biomimetic applications. To the best of our knowledge, no comprehensive review of the existing literature on this topic has been published. Furthermore, the field is disjointed due to the use of various terms to describe plant CW-like materials. The terms “artificial”, “synthetic” plant CWs, wall-like materials, wall-mimicking materials, and in vitro grown CWs are all used interchangeably to describe plant CW-like materials in the literature. In this paper, we aim to consolidate the current knowledge of this field by reviewing key studies on native plant CWs and CW-like materials. After providing a general description of plant CWs, including their composition and micro-/nanoarchitecture, we review the fabrication methods and characterization techniques for CW-like composites. Finally, the emerging applications of these materials are reviewed.

## Plant CWs: Function, Architecture, and Composition

**Fig. 1:**
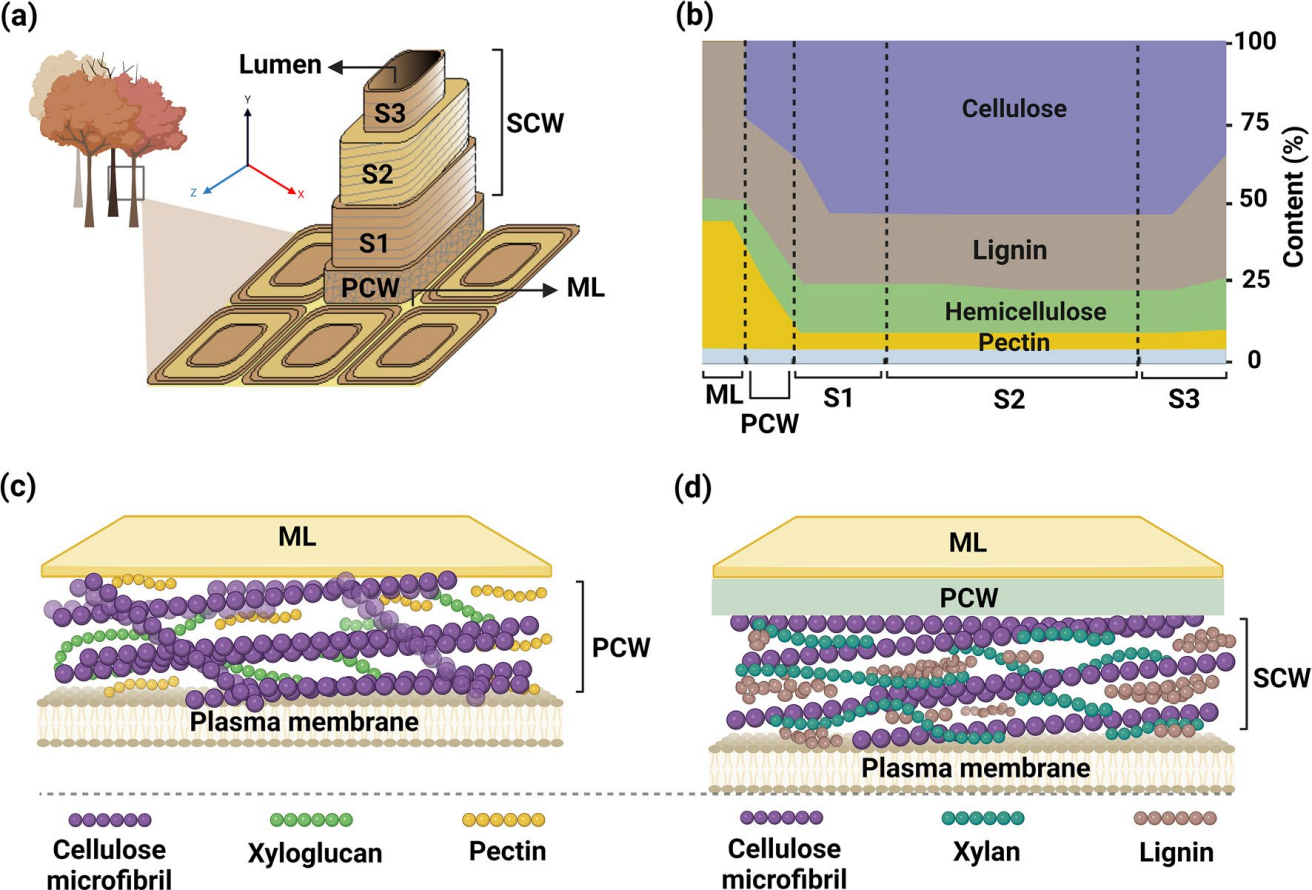
CW architecture and composition. **a** A cut-out schematic of a wood CW shows its layered structure, featuring a network-like arrangement in the PCW and aligned fibrils in the SCW. **b** The composition of wood CW in each layer after lignification. The light blue region shows the content of other compounds in the layers. **c** PCW schematic based on a molecular model, showing a load-bearing network of CMF in a matrix of pectin and xyloglucan. **d** A schematic of the SCW based on a molecular model, illustrating lignin deposits within an oriented cellulose matrix. The CMF are bound by xylan hemicelluloses, with limited interactions between cellulose and lignin. The PCW and SCW schemes were inspired by [29] and [37], respectively

The hierarchical structure and constituent elements of plant CWs are discussed herein to provide a fundamental understanding of how CWs may be mimicked. The main function of CWs is to provide structural resilience and protect cells against biotic and abiotic stresses, while passing nutrients, gases, and cellular signals to the plasma membrane. CWs must be strong to support the mechanical integrity of plants and be extensible to enable cell expansion in growing cells [4, 5, 18]. All plant cells have a strong, extensible CW layer called the primary cell wall (PCW), while specialized cells, such as tracheary elements and wood fibers, encompass an additional secondary cell wall (SCW). Figure 1a presents the layers of CW in wood, including the middle lamella (ML), PCW, and SCW, as well as lumen. The SCW is composed of three sublayers, namely S1, S2, and S3, separating SCW from the lumen (i.e., the central void space). Each layer has unique arrangement of cellulose microfibrils (CMF), which play a key role in defining the physical and mechanical properties of plant CW [19]. The lumen in tracheary elements is responsible for water transport, and the CWs between adjacent cells are adhered to each other by the pectin-rich ML [3]. Both PCW and SCW layers contain CMF, which are frequently bundled together to provide much of the structural strength for the CWs [1, 4].

The precise composition of CWs varies between species and even among the specialized tissues within the same plant [20]. Figure 1b presents the variations in composition and the corresponding content within each layer of a lignified CW in wood. It is important to note that these are final compositions after the cell growth is ceased, and the actual compositions during the cell growth are different because not all components are synthesized and deposited at the same time. The PCW is a composite material with both elastic and plastic properties, consisting of three structural polysaccharides, namely CMF (otherwise known as cellulose nanofibrils or CNF) (~ 15%-40%), hemicelluloses (~ 20%-30%), and pectin (~ 30%-50%) [2]. In lignified cells, the PCW also contains lignin (e.g., tracheid cell, up to 70%) [21]. The predominant hemicelluloses are xyloglucans in the PCW of all spermatophytes except grasses [22]. The SCW, which is formed after the plant cells stop growing, is thicker than the PCW. This layer consists of cellulose (~ 40%-48%), hemicelluloses (~ 20%-30%), and lignin (~ 20%-30%) [23]. In addition to the dominant structural polymers, growing CWs comprise small amounts of glycoproteins, also called structural proteins; however, they do not significantly contribute to the mechanical properties of CWs [18].

Information on the morphological features of plant CWs is being continuously updated, as current studies are limited to nonliving plant materials and in silico modeling [24–26]. The architecture of CWs depends on the orientation/arrangement of structural components [27, 28]. Figure 1c presents a recent model for PCW, consisting of a cross-lamellate cellulose fibril network embedded in a polysaccharide matrix [29]. Hemicellulose chains noncovalently bind to CMF, while the hydrated pectin molecules form a gel-like matrix, filling the space among the stiff cellulose networks. Since the PCW is formed during the growth phase of plant tissues, it must be extensible and capable of expanding alongside the growing plant cells [2, 5, 30]. During cell expansion, turgor pressure (i.e., the force from within the cell pushing the membrane against the CW) generates wall stresses and stores mechanical energy within the cell. Mechanical creep allows wall polymers to relax, facilitating the controlled expansion of CW. The stored mechanical energy drives cell expansion. This process is aided by wall-loosening agents, such as expansins, which disrupt crosslinks between CMF, allowing the fibrils to slide past each other more easily [4]. Additionally, plant cells are adhered to one another by a pectin-rich layer known as the ML [3].

Compared with PCW, SCW is stiffer and supports the plant weight; it is formed after the cell ceases to grow. It provides compressive and tensile strength, but not necessarily extensibility [2]. In cells containing a SCW, the PCW and ML become increasingly lignified, which are referred to as a compound middle lamella (CML) [21]. In the SCW S1 and S3 layers, CMF align roughly perpendicularly to the longitudinal axis of cell (*Y* axis, shown in Fig. 1a) [21, 31]. In the S2 layer, comprising the majority of CW, highly oriented CMF are directed within a specified angle (*Z* axis, shown in Fig. 1a), known as microfibril angle (MFA) [32–35]. Figure 1d presents a recent model of SCW, where hemicellulose binds to the CMF, and lignin is bridged to CMF through hemicelluloses, indicating that CMF and lignin do not interact directly [2]. Xylan and glucomannan (GM) are two of the most prevalent hemicelluloses in the SCW [23]. Lignin, composed of phenolic compounds, imparts strength, rigidity, and hydrophobicity to the SCW. Its composition can vary as a result of the random co-polymerization of coniferyl, syringyl, or p-coumaryl alcohols, known as monolignols, which are synthesized in the cytosol and transported to the CW. Once in the CW, these monolignols are polymerized through oxidation, facilitated by oxidase enzymes such as peroxidase [23, 36]. The deposition of lignin and hemicellulose renders the water conduits waterproof, enabling efficient water transport throughout the plants and increasing the stiffness and strength of CW [23, 37].

## Fabrication and Properties of Artificial Plant CWs

**Fig. 2:**
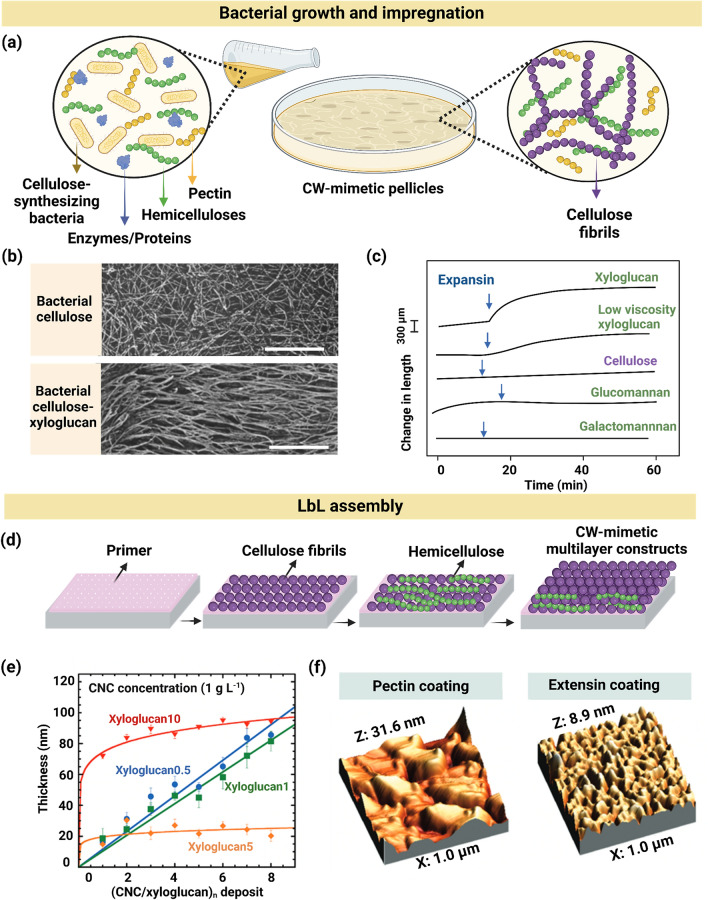
Fabrication of plant CW-like materials via bacterial cellulose pellicle growth and impregnation or LbL assembly techniques. **a** A culture of cellulose-synthesizing bacteria, assembling cellulose within a growth medium, containing CW polysaccharides and other biopolymers. **b** Deep-etch freeze-fracture TEM micrographs of bacterial cellulose, showing differences in the arrangement of CMF with or without xyloglucan. Scale bars are 5 µm. Adapted (cropped and labeled) with permission [46]. Copyright Wiley, 1995. **c** Effect of expansin on the extension of hemicellulose-cellulose composites, showing a significant increase in displacement under a constant load for cellulose-xyloglucan composites with the addition of expansin. Adapted with permission. Copyright Wiley, 2000 [39]. **d** Schematic of the LbL assembly method, showing the formation of alternating cellulose fibril and xyloglucan layers on a primer-coated substrate. **e** The thickness of CNC-xyloglucan films, formed via spin coating, as a function of deposited layer number demonstrates the linear growth of layers when the xyloglucan concentration is 0.5 or 1 g mL^−1^. Adapted with permission [62]. Copyright American Chemical Society, 2010. **f** AFM topographical images show the non-uniformity of pectin and extensin layers in a pectin-extensin composite material, attributed to the imbalanced charge density between the components. Adapted with permission [65]. Copyright American Chemical Society, 2010

**Fig. 3:**
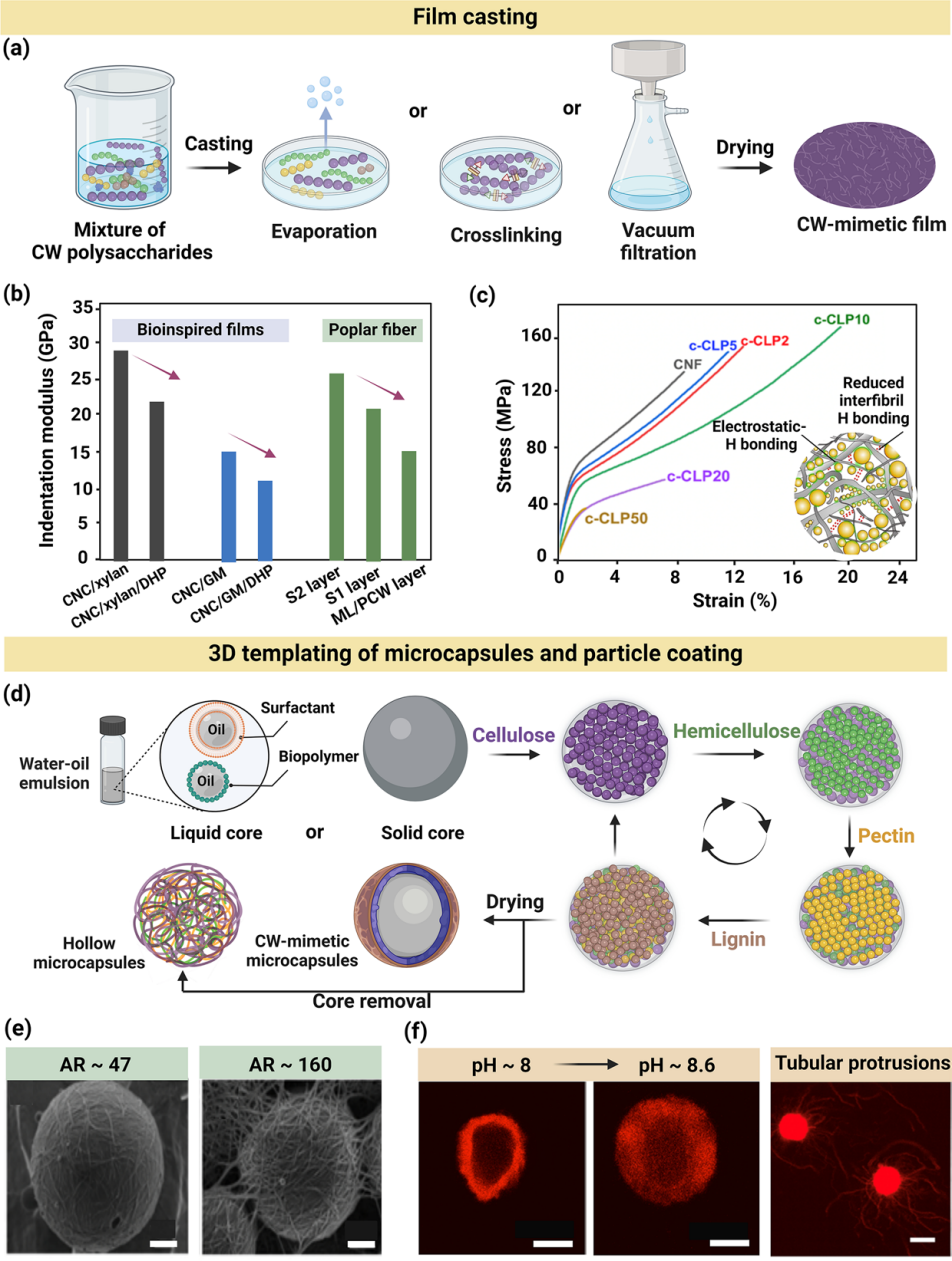
Fabrication of plant CW-like materials via film casting and 3D templating techniques.** a** Schematic of the film casting technique for constructing a plant CW-like film by combining CW polysaccharides, followed by solvent removal through either evaporation, crosslinking, or filtration. **b** Nano-indentation modulus of in situ lignified cast films, showing that an increase in lignin content and a decrease in cellulose content reduced stiffness. Adapted under terms of the CC-BY license [69]. Copyright 2017, The Authors, published by Springer Nature. **c** Tensile stress–strain of cast films, containing c-CLPs, showing an initial increase in both strength and elongation at break by increasing the lignin content (e.g., tensile strength at break increases from 132 to 160 MPa as c-CLPs increased from 0 to 10%), followed by a rapid decrease for films containing 20% and 50% c-CLPs [74]. Values next to c-CLP on the curves indicate the lignin content (wt%) of films. H bonding stands for hydrogen bonding. Adapted under terms of the CC-BY license [74]. Copyright 2019, The Authors, published by American Chemical Society. **d** LbL assembly of CW-mimetic microcapsules using liquid- or solid-based 3D templating and particle coating techniques, resulting in hollow microcapsules when the core was removed. **e** SEM images of microcapsules composed of CNC, isolated from bacteria or algae with varying AR, showing differences in the morphology of droplets and inter-droplet bridging. Core spheres are polystyrene particles, representing the oil droplets. Adapted with permission [85]. Copyright Royal Society of Chemistry, 2013. **f** Plantosomes prepared using liposome templating mimicked the mechanism of turgor pressure in native plant cells by undergoing reversible deformations and the formation of microtubular protrusions when the pH was increased from 8 to 8.6. Adapted under terms of the CC-BY license [87]. Copyright 2020, The Authors, published by Springer Nature

Artificial plant CWs have been constructed via bottom-up approaches using bacterial cellulose pellicle growth and impregnation, layer-by-layer (LbL) assembly, film casting, 3D templating microcapsules, and particle coating. These methods, summarized in Figs. 2 and 3, are discussed in this Section.

### Bacterial Pellicle Growth and Impregnation

Bacterial cellulose has high purity and degree of polymerization (DP), whereas mechanically fibrillated and chemically treated cellulose isolated from plant sources typically have lower or less uniform DP [38]. Leveraging these advantages, bacterial cellulose pellicles are cultivated, and secondary components are impregnated into the pellicles to construct artificial plant CWs using a bottom-up approach. To achieve this, cellulose-producing bacteria are cultured in media containing the constituent components of plant CWs, facilitating in vitro cellulose assembly that mimics the structures of natural CWs. Bacterial cellulose is synthesized as large, randomly oriented ribbons, which has been used to simulate the biomechanical properties of CWs, such as extensibility, as well as physicochemical phenomena like water sorption kinetics [10, 39, 40]. Although artificial CWs can provide valuable insights, they exhibit significant structural differences compared with native plant CWs, where CMF are considerably thinner [41]. Moreover, native plant CWs are oriented in biologically determined patterns, identified by MFA, which contrast sharply with the random 3D weave of cellulose ribbons in bacterial pellicles. These differences result in distinct mechanical properties between the two materials [42, 43]. MFA, describing the angle between the CMF and the long axis of the cell, plays a crucial role in determining the mechanical properties of plant tissues, particularly in how they respond to stress and strain, influencing properties such as stiffness, flexibility, and resistance to bending or stretching [44, 45].

Figure 2a shows a representative culture of cellulose-producing bacteria within a growth medium, enriched with CW polysaccharides and other biopolymers. This method was initially demonstrated in a series of studies by Whitney et al., [10, 39, 46, 47] in which bacterial cellulose pellicles were assembled in hemicelluloses (e.g., xyloglucan and GM), followed by the addition of expansins (nonenzymatic proteins that mediate CW loosening and assist in extensibility). This technique was used to investigate how xyloglucan and GM bind to CMF and contribute to the formation of CMF networks within CWs. In a similar study based on bacterial cellulose, *Gluconacetobacter xylinus* (formerly *Acetobacter xylinum*) was grown in a hemicellulose-containing medium. The addition of xyloglucan to the culture media resulted in a preferential alignment of fibrils, which was attributed to the formation of crosslinks between bacterial CMF through hydrogen bonding. Figure 2b shows the transmission electron microscopy (TEM) micrographs of bacterial cellulose composites formed in the absence or presence of xyloglucan. The xyloglucan backbone, adopting a cellulose-like conformation, supported its binding to cellulose and facilitated CMF alignment [46]. Galactomannan (0.2 w/v%) and GM (0.5 w/v%) similarly underwent crosslinking with CMF and exhibited alignment, with a tendency to self-aggregate at higher galactomannan concentrations. This behavior is attributed to concentrations significantly exceeding the experimental entanglement concentration of approximately 0.23 w/v% for galactomannan [47]. The increased entanglement may cause polymers that do not normally bind to cellulose to become trapped within the cellulose networks.

To more closely mimic native CWs compared with composites containing only bacterial cellulose and hemicellulose, CW proteins (e.g., expansins) and other CW biopolymers (e.g., pectin) should be used to enhance extensibility, reflecting the properties of native plants. “Extensibility” refers to the ability of native CW or plant CW-mimetic materials to expand under a constant force due to the action of wall-loosening agents, which should not be confused with or used interchangeably with the extensibility of CWs under increasing tensile stress [48]. This property is crucial for artificial CW materials to replicate the ability of plant CWs to undergo extension, which is induced and mediated by expansins during cell growth. The effect of CW proteins on the mechanical properties of CW-mimic materials was examined via adding α-expansin (CsExp1) to hemicellulose-bacterial cellulose composites [39]. Figure 2c presents the effect of CsExp1 on the extensibility of hemicellulose-cellulose composites as a function of time. Here, extensibility was quantified by the extension rate: the rate of extension measured shortly after the addition of expansins, minus the rate observed before expansin addition [48]. Expansin increased the extensibility of cellulose-tamarind xyloglucan composite to a greater extent (96.3 µm min^−1^) compared with cellulose alone (0.4 µm min^−1^) and cellulose composites containing low viscosity xyloglucan (24.8 µm min^−1^), GM (~ 5.3 µm min^−1^), or galactomannan (~ 0.5 µm min^−1^). This was attributed to the longer chain and higher molecular weight of tamarind xyloglucan, resulting in the formation of more crosslinked domains in the bacterial cellulose composites.

Several studies suggest that the dual digestion of cellulose and xyloglucan induces CW expansion, indicating the presence of a mechanical ‘hotspot’ involving both cellulose and xyloglucan [39, 49, 50]. Inspired by this mechanism, *Gluconoacetobacter xylinus* was used to fabricate CW-like composites of xyloglucan-cellulose and pectin-xyloglucan-cellulose. Xyloglucan, in the presence of cellulose or pectin-cellulose, formed compliant materials with a time-dependent creep behavior within biaxial constraints. When pectin was incorporated into the material, the material strength and stiffness decreased while the ultimate strain (i.e., the maximum strain before failure) increased. The decrease in modulus and increase in extensibility were attributed to changes in the architectural organization of CMF upon pectin addition, which resulted in greater inter-fibrillar freedom [51]. The *Gluconacetobacter xylinus* has also been used to assemble artificial apple CW-like composites by culturing the bacteria in a medium containing apple pectin and xyloglucan [8]. The composite was compared with other CW analogs fabricated by mixing components such as bacterial cellulose, pectin, and xyloglucan. The composites produced from culturing bacterial cellulose in the presence of pectin and/or xyloglucan had mechanical properties more closely resembling native plant tissues. The bacterial cellulose-xyloglucan-pectin composites also had higher ultimate strain at a high relative humidity (i.e., ~ 95%), as the failure strain increased from ~ 0.05 in the homogenized/mixed samples to ~ 0.3 in the cultured bacterial cellulose-pectin-xyloglucan composites, which more closely reflects the extensibility of plant tissues (~ 50%) [42, 52]. The extensibility of the artificial CW-like film at high relative humidity was attributed to the hydration of pectin, facilitating the movement of CMF network under extension [8]. In a subsequent study, calcium ions were added to the bacterial cellulose-pectin-xyloglucan composite to resemble apple CW and study the effect of calcium ions on the composite mechanical properties. The effect of calcium on mechanical properties was analogous to that observed in the apple tissue, where pectin chains were crosslinked by divalent ions. In plant CW-like composites containing pectin, calcium crosslinked the pectin chains, as evidenced by an increase in failure stress from ~ 0.7 to 1 MPa when the calcium concentration was increased from 0 to 8% w/v [53].

The studies discussed above demonstrate that bacterial cellulose may be cultured in media containing various CW components to explore polymer interactions and assembly in complex CW-mimetic composites. As additional components are incorporated (e.g., biopolymers, expansins, calcium ions), the mechanical properties more closely mimic those of plant CWs under realistic conditions, such as high relative humidity. To better understand the interactions and binding kinetics of CW constituents, three hemicelluloses (i.e., xyloglucan, water soluble xylan, and galactoglucomannan) were adsorbed to in vitro-grown bacterial pellicles, and isotherms were obtained based on the equilibrium concentration of adsorbed hemicelluloses. Xyloglucan had the highest affinity for cellulose. Additionally, the hemicellulose-bacterial cellulose pellicle films were lignified by depositing coniferyl alcohol monolignols onto their surface, followed by polymerization mediated by horseradish peroxidase (HRP) and hydrogen peroxide. Xyloglucan and water-soluble xylan enhanced lignin formation on their respective cellulose matrices by facilitating the formation of aryl ether and 5–5' interunitary linkages, respectively. In contrast, galactoglucomannan inhibited lignin formation due to steric repulsion between the galactosyl substituents and the monolignols [54].

In CW-like materials, tailoring the interactions of CW components for water absorption and transport is important to mimic realistic plant conditions [55]. *Gluconoacetobacter xylinus* bacterial pellicles have been used to study the binding kinetics of polysaccharides to CMF, as well as to investigate how different compositions affect the water sorption–desorption kinetics of CW-like materials [40, 54]. CW-like composites made up of varying amounts of bacterial cellulose pellicles, pectin, and xyloglucan were fabricated to gain a better understanding of water exchange properties in native fruit CWs. The data collected were fitted to water absorption models, including Standard Guggenheim-Anderson-de Boer (GAB) and Ferro-Fontán [56–58]. From these models, the water retention capacity (the amount of water adsorbed and/or absorbed by the composite), water conductivity (the rate at which water molecules move through the composite), and diffusivity (the rate at which water molecules spread evenly through the composite) in the artificial CW were measured. The addition of pectin to the culture media reduced cellulose bundle size and the porosity, resulting in a denser fibril network compared with pure cellulose. This decreased the water retention capacity (~ 8.16 to ~ 6.0 kg_water_ kg_dried_^−1^ Pa × 10^–8^), water conductivity (~ 17.3 × 10^–16^ to ~ 11.9 × 10^–16^ kg m^−1^ Pa^−1^ s^−1^), and diffusion coefficient (~ 48.8 × 10^–11^ to ~ 46.8 × 10^–11^ m^2^ s^−1^) at 25 °C and 85% relative humidity. These values further decreased upon xyloglucan addition to the cellulose-pectin composites [40]. The significance of this study lies in the application of CW-like materials as a barrier to water exchange, inspired by the barrier properties of plant CWs, which helps preserve the moisture of plant-based products during commercial storage.

### Layer-by-Layer Assembly Technique

Via dip coating, spin coating, or pipetting LbL assembly, alternating layers of plant CW components have been deposited on a variety of substrates (e.g., charged, metal, or biopolymeric surfaces). The precision of this technique in controlling material structure has enabled the creation of heterogenous domains within CW-like bulk composites [59]. Figure 2d presents the LbL assembly method via which alternating layers of CMF and xyloglucans are deposited on a primer-coated substrate. Depending on the intended application of CW-like materials, CMF may be micro- or nano-engineered prior to assembly to incorporate specific functional groups or alter their morphology. In LbL studies, the substrate is first coated with a polyelectrolyte multilayer primer to ensure a uniform coating and minimize the effects of substrate composition and texture before depositing the first layer of materials [60]. This is because the LbL technique typically relies on charged polymers to enable interlayer adhesion; however, some studies have also reported the construction of multilayered films using only hydrogen bonding or van der Waals forces [14, 61, 62]. Accordingly, CW-like films of alternating thin layers of cellulose nanocrystals (CNC) and xyloglucans were constructed via non-electrostatic interactions [62, 63]. CNC-xyloglucan films produced by spin coating were more uniform compared with the analogs generated by dip coating. At a constant CNC concentration (1 g L^−1^), concentrated solutions of xyloglucan (e.g., 5 or 10 g mL^−1^) could only build 2–4 bilayers as a result of polymer entanglement, after which the film thickness plateaued. In contrast, dilute xyloglucan solutions (e.g., 0.5 or 1 g mL^−1^) allowed for the formation of an unlimited number of bilayers. Figure 2e presents the thickness of CNC-xyloglucan films formed by spin-coating as a function of the number of deposited layers, confirming the linear growth of layers for composites containing 0.5 and 1 g mL^−1^ of xyloglucan [62].

As mentioned, charged biopolymers such as pectin are particularly advantageous for the LbL technique [64, 65]. The balance between the biopolymer charge densities is crucial in determining layer organization and architecture. Using electrostatically driven LbL assembly, pectin-extensin films were fabricated on silicon wafers. Extensins are positively charged hydroxyproline-rich glycoproteins, existing in the CWs of higher plants [66]. Since pectin and extensin have opposite charges, each layer adheres to the adjacent layer through electrostatic interactions. A non-uniform growth of the pectin-extensin LbL film was observed, resulting from diffusive mixing at the interface of the polymer layers, as confirmed by atomic force microscopy (AFM) topographical images (Fig. 2f). It was theorized that the film non-uniformity resulted from extensin being weakly charged while pectin was strongly charged. This imbalance in charge density led to non-linear growth of the pectin and extensin layers [65]. In both linear and non-linear growth of layers, LbL assembly has facilitated the investigation of interactions between specific components (e.g., cellulose and xyloglucan, pectin and extensin) in WC-like materials.

LbL assembly has also been adopted to study the interactions (particularly non-covalent) between CW polysaccharides and lignin model compounds in CW-like materials. Lignified CNC films were prepared using a dehydrogenation polymer (DHP), a model lignin derived from the polymerization of monolignols with hydrogen peroxide and peroxidase, to mimic wood CW lignification [67]. The DHP was prepared via the “Zutropfverbaten” method, entailing the slow, continuous coniferyl alcohol and hydrogen peroxide addition to a peroxidase solution, and was spin coated alternatively with CNC. The AFM topographical analysis confirmed that the DHP formed globular structures, which helped CNC adhere to the coating. Spectroscopic ellipsometry, along with classical or Tauc-Lorentz model fitting, were used to measure DHP thickness on CNC films. The apparent layer thickness of DHP increased from ~ 7.5 to ~ 141 Å after 6 h of contact, followed by a decrease to ~ 80 Å after rinsing with water and drying. This suggests non-covalent interactions between DHP and CNC, primarily through hydrogen bonding and hydrophobic interactions [67]. Together, LbL techniques have enabled the precise investigation of interactions between two CW components, which would be challenging to achieve in systems with more complex compositions.

### Film Casting

Film casting is a scalable and biologically relevant technique for fabricating CW-like materials. This method involves mixing several components of interest in a solution, followed by casting the mixture into a film, which allows for the investigation of interactions among CW components, particularly their effects on mechanical, morphological, and hygroscopic properties [68, 69]. Castings may be solidified by evaporation or filtering out the excess solvent, followed by physical and/or chemical crosslinking [70–74]. Figure 3a presents the construction of a plant CW-like film via casting wherein the solvent is either evaporated or filtered. Film casting has been used to visualize biomass recalcitrance in situ, specifically examining the effect of lignin content on cellulose accessibility [68, 69]. To investigate the impact of lignin content on its recalcitrant behavior during enzymatic hydrolysis, suspensions of lignin and CNF (0.4 wt%) were prepared, and the lignin concentration was increased up to 40 wt%, followed by casting the mixtures in Petri dishes. Small pieces of films were submerged in a Cellic CTec2 enzyme solution (0.5 wt%) at 40 °C for 2 h. Increasing lignin content significantly impeded enzymatic cellulose hydrolysis because the CNF were embedded within the lignin matrix, restricting their accessibility, which was observed through the in situ AFM analysis of film morphology [68].

Artificially lignified films have also been fabricated via film casting for SCW studies [69, 70, 72]. Muraille et al., constructed films from a single polymer (CNC, hemicellulose, or DHP), binary polymers (hemicellulose/CNC, DHP/CNC, or DHP/hemicellulose), and ternary polymers (DHP/hemicellulose/CNC) [69, 70]. The films were either lignified by the in situ surface polymerization of DHP or mixing with lignin directly, followed by casting. As opposed to in situ surface polymerization, the mixing method led to higher film heterogeneity due to the polysaccharides-DHP phase separation, as well as water evaporation during film formation. Notably, the in situ polymerized films were more hygroscopic than the cast films of lignin-polysaccharide mixture. Compared with the simple mixing-casting method by which lignin hydrophobicity was well expressed, in situ DHP polymerization resulted in more interactions between lignin and polysaccharides (e.g., CNC and hemicellulose), limiting the lignin contribution to hydrophobic interactions. Besides lignin, the composition and ratio of CNC and hemicelluloses had significant impacts on the hygroscopic properties of lignified films as a result of varying affinities between lignin and the CW polysaccharides. Accordingly, the increase in the water adsorption and retention of in situ polymerized films was attributed to the covalent bonds formed in the hemicellulose-DHP during the polymerization process [70].

In another study, lignified composites were fabricated via film casting using varying ratios of CNC, hemicellulose, and DHP to analyze composite mechanical properties. Figure 3b presents the indentation moduli of bioinspired films (i.e., CNC/xylan, CNC/xylan/DHP, CNC/GM, and CNC/GM/DHP) and natural fibers. The indentation moduli of CNC/hemicellulose/DHP (4/1/1 ratio) films were within the same order of magnitude as the CW of natural fibers (15–25 GPa). Films composed of CNC/GM/DHP or CNC/xylan/DHP had an indentation modulus of 11 or 22 GPa, respectively. All lignified films underwent a decrease in modulus compared with lignin-free films, except for the CNC/DHP films (not shown in Fig. 3b, ~ 55 GPa). For instance, the modulus of CNC/xylan films decreased from 28 to 22 GPa by DHP addition. The decrease in mechanical properties was proportional to the reduction in CNC content in lignified ternary films (~ 66% CNC) compared with binary films (~ 80% CNC), which indicated that a higher cellulose content mitigated the impact of lignin on the mechanical properties [69]. Several studies have highlighted that reactive functional groups of cellulose (e.g., hydroxyl groups) and its surface roughness facilitate electrostatic and van der Waals interactions with both hydrophilic and hydrophobic polymers, such as lignin [75, 76], likely resulting in the improved mechanical properties.

Since the interactions between cellulose and lignin depend on lignin morphology, film casting has also been used to investigate the effect of lignin morphology within lignocellulose matrices on the mechanical characteristics of films. Figure 3c presents tensile stress–strain curves of nanocomposite films, containing varying contents of cationic spherical colloidal lignin particles (c-CLP). While many studies have reported that film stiffness and strength decrease as lignin content increases, incorporating ~ 10 wt% of c-CLPs (hydrodynamic diameter ~ 102 nm) in CNF films increased the strength at break and toughness compared with pure CNF films. The well-defined spherical morphology of c-CLPs allowed lignin to fill the interstitial areas and void spaces, leading to effective stress transfer to the CMF and increased ductility and toughness. This feature closely resembles the biological role of lignin in natural plant CWs, where it supports the interconnection of cellulose network. In contrast, in other composites, lignin typically forms large aggregates that disrupt the interfibrillar hydrogen bonding within cellulose networks [75]. To mimic the covalent bond formation in lignin-carbohydrate complexes (LCC) within plant CWs, the casting method was used to form lignocellulosic composite films and hydrogels via physical entanglement or epichlorohydrin-mediated chemical crosslinking. Films and hydrogels were prepared from carboxyl-modified CNF, alkali lignin, and GM as a compatibilizer. Both types of crosslinks similarly altered physical and mechanical properties: strength and stiffness decreased, while hydrophobicity increased by increasing the lignin content. However, films with LCC-mimicking bonds had greater stiffness (and higher hydrophobicity) compared with those with physical crosslinks, with Young’s moduli of approximately 10.1 and 7.6 GPa, respectively [73].

Film casting has also been used to study the interactions between cellulose and hemicelluloses, which have an opposite effect on mechanical properties compared with lignin [71, 77]. CNF-hemicellulose films were prepared using three types of CNF with similar dimensions and varying hemicellulose content, namely, holo-CNF (i.e., CMF bearing a high hemicellulose content), enzymatically-treated CNF, and carboxyl-modified CNF, prepared via 2,2,6,6-tetramethylpiperidine 1-oxyl (TEMPO)-mediated oxidation. The mechanical properties of films composed of chemically treated fibrils were compared with those made from fibrils subjected to less intensive mechanical processing. The mechanical processing which preserved a high amount of hemicellulose on the CNF resulted in films with the highest strength compared with those made with carboxyl-modified and enzymatically treated CNF. This trend was evident even at high relative humidity levels (i.e., 50% or 90%). Dry, holo-CNF films had a Young’s modulus of 24.9 GPa and ultimate strength of 348 MPa. Notably, these films were much stronger than the ones composed of biaxially oriented polyethylene terephthalate. The remarkable mechanical properties of holo-CNF films were attributed to: (i) the intrinsic high modulus and strength of holo-CNF as a result of mild isolation processing and (ii) well-preserved hemicelluloses surrounding the nanofibers, increasing interfibrillar bonding and stress transfer among the fibrils [77].

### 3D Templating Microcapsules and Particle Coating

To fabricate synthetic plant CWs or tissues, one approach involves subjecting biopolymers to more geometrically relevant 3D constructs [78–81]. Coating of polysaccharides such as dextran, pullulan, and hemicelluloses onto liposomes [78, 79] and cells [81] originated in the early 1990s. Figure 3d presents the construction of CW-mimetic constructs via liquid- or solid-based 3D templating of microcapsules and particle coating in a LbL fashion. CW-like core–shell particles have been developed using two distinct 3D templating strategies: (i) liquid cores, where the interior space of capsules is filled with either an emulsion or an aqueous phase, and (ii) solid cores, where colloidal particles are used as templates for the deposition and assembly of components. In the latter case, the core may be removed to generate a hollow polymeric shell. The solid templates may be silicon dioxide or carbonate microparticles such as manganese, calcium, or cadmium species [80], or crosslinked polymers (e.g., polystyrene) [82].

The solid core templating approach has been used to construct synthetic multilayer capsules based on electrostatic interactions. Inspired by the structure and composition of plant PCW, 3D templated solid-core microcapsules (diameter ~ 16 µm) were fabricated using calcium carbonate as a template and plant polysaccharides (e.g., cationic CNF, pectin, and xyloglucan) as a shell [80, 83]. The calcium carbonate particles were treated with citric acid (50 mM) for 3 h to generate hollow microcapsules. The microcapsules had properties that mimic those of plant CWs, particularly in terms of permeability and mechanical stability. The barrier properties of microcapsules were examined using fluorescein isothiocyanate (FITC)-labelled dextran molecules with varying hydrodynamic sizes (e.g., 6.6, 12, or 54 nm). In water, only dextran molecules with a hydrodynamic diameter of 6.6 nm could penetrate the capsule wall, made of apple pectin, xyloglucan, and CNF. The wall consisted of a percolating network of CNF, with xyloglucan and pectin located among the nanofibers. When exposed to saline (NaCl, 10 mM), larger dextran molecules with a hydrodynamic diameter of 12 nm also permeated the capsule wall. The results indicated that capsule walls were responsive to saline, becoming more porous and permeable as a result of a salt-mediated decrease in the hydrodynamic size of pectin. The subsequent uptake/release of molecules using the same microcapsule for multiple cycles was possible by changing the media from water to saline and vice versa. Microcapsules containing water-soluble pectin had high integrity and robustness after multiple cycles of salt loading/unloading, which was attributed to the percolating CNF networks [80].

The permeability of microcapsules is tunable via tailoring the capsule wall composition. To investigate this effect, two types of microcapsules were assembled using 5 bilayers of cationic CNF and xyloglucan, with and without pectin. Increasing the xyloglucan and decreasing the pectin contents resulted in microcapsules with decreased permeability in saline solutions. Although the microcapsules in water were permeable to all sizes of FITC-dextran molecules (i.e., 6, 12, and 54 nm), only the 6 nm dextran molecules and a small fraction of the 12 nm molecules permeated into the microcapsules in saline. The opposite permeability of this microcapsule in water with the above-described one was explained by the citric acid-mediated physical crosslinking of capsule wall, decreasing the swelling degree and pore size. In saline, the low permeability of cationic CNF/xyloglucan/pectin microcapsules was attributed to the electrical double layer (EDL) screening of charged polymers by NaCl, allowing nonionic interactions such as hydrogen bonding and van der Waals attraction among CNF. As a result, the capsule wall thickness decreased, resulting in the decrease in pore size [83].

Liquid core templating has also been used to create CW-mimetic constructs. This approach was inspired by the assembly of nanocelluloses in Pickering emulsions. In 2011, varying concentrations of bacterial CNC (0 to 5 g L^−1^) were used to stabilize hexadecane-water emulsions, forming liquid-core microspheres (diameter ~ 4 μm). The CNC-coated oil-in-water droplets were stable at ambient condition for several months [84]. The droplet stability was associated with the irreversible interfacial adsorption of CNC and the formation of 2D cellulosic network at the interface. This study was further expanded by the same group to investigate how CNC origin and dimensions affect the stability of liquid core systems (i.e., oil-in-water emulsion) [85]. Particle aspect ratio (AR) had a significant impact on droplet surface coverage. Figure 3e presents scanning electron microscopy (SEM) images of the microspheres coated with CNC (AR ~ 47 or 160). Short CNC (length ~ 189 nm, AR ~ 47) densely coated the droplets with a surface coverage of more than 80%, whereas long nanocrystals (length ~ 4000 nm, AR ~ 160) formed a loose interconnected network with a reduced coverage of ~ 40% [85]. The high coverage achieved by shorter CNC was attributed to their ability in aligning side by side via the strong and long-range capillary attractive forces. However, obtaining uniform alignment throughout the droplet is challenging for elongated nanocrystals with a high AR.

To mimic the architecture and properties of fruit and vegetable parenchyma cells, liquid-core capsules with high mechanical strength were fabricated using a blend of short CNF (length < 1 µm) and CNC (length ~ 140 nm) via the Pickering mechanism. A crosslinked network of CNF-CNC was formed at the interfaces using isophorone diisocyanate, wherein a urethane bond was generated by reacting cellulose hydroxyl groups with isocyanate. In this reaction, urea bonds were also formed via reacting diisocyanate groups with water. As a result, the outer and inner layers of shell wall structure consisted of covalently crosslinked CNF-CNC and aromatic polyurea, respectively. Combining short CNF and CNC favored a close-packed arrangement, resulting in capsule walls with a high cellulose content. When the total dry content of CNF/CNC was 17 wt%, the capsules attained an indentation modulus of ~ 4.84 GPa, which was 6 times higher than that of polyurea capsules (~ 0.79 GPa) and 3 orders of magnitude higher than that of capsules composed of dissolved cellulose (~ 0.0074 GPa). The high modulus was attributed to the close-packed structure of capsule wall outer layer, which was formed by the oriented CNF and CNC. This structure was reinforced by both covalent bonds (between the urethane matrix and CNF/CNC) and hydrogen bonds (between CNF and CNC or between the urethane matrix and CNF/CNC). The wall structure of capsules closely mimicked the CW of seedling stem cells in embryonic cucumber [86].

In another study, more complex microcapsules that mimicked both plant CW and membrane were fabricated via liquid-core 3D templating. The outer shell of microcapsules consisted of PCW polysaccharides, including chemically modified cationic CNF and pectin, and the inner shell consisted of a thin layer of lipids, including oleic acid, oleate, and structural plant phospholipids surrounding a water core [87]. The microcapsules (diameter ~ 27 µm) with a plasma membrane-like core, covered by a continuous fiber layer, were referred to as plantosomes. At pH > 7, oleic acid may self-assemble into organized structures including vesicles, micelles, and cubic phases [88]. By leveraging the pH-dependent phase behavior of lipids, plantosomes with vesicle-rich cores were developed to mimic the expansion observed in native plant cells during growth. Plantosomes absorbed water at a final pH of 8.6 in an ammonium acetate solution, a solute known to enhance the permeability of vesicle membranes. The water uptake was also attributed to the pH-mediated self-assembly of interior oleic acid/oleate/phospholipids mixture into vesicles, increasing the plantosome radius and surface area. Figure 3f shows the plantosome radius change (~ 29%) by increasing pH, mimicking the physical expansion of plant cells. Notably, this is distinct from plant cell growth, which occurs when pH decreases. During expansion, the lipid molecules diffused from the core toward the outer shell, generating tubular structures that stretch across the plant CW-like shell, as shown in Fig. 3f. The formation of lipid tubular protrusions was attributed to the restrictive, cage-like CNF/pectin wall, which limited indefinite expansion, allowing the lipids to protrude only through the pores of polysaccharide wall. Upon the addition of magnesium ions (10 mM), the expanded plantosomes collapsed as a result of ion-mediated bridging and the merging of smaller vesicles into larger ones, causing the protrusions to disappear [87].

Through a similar liquid core templating strategy, CW-mimetic microcapsules were fabricated using alternating layers of pectin, xyloglucan, and CNC, deposited on giant unilamellar vesicles (GUV) via LbL assembly. The average diameter of microcapsules with 10.5 layers of polysaccharides was ~ 25 µm. Upon glucose-mediated osmotic shocks, the microcapsules underwent a significant reversible deformation as a result of shell elasticity. The GUV membrane (i.e., shell) permitted the microcapsules to expand and contract in response to the osmotic pressure changes, leading to the reversible shape changes. The indentation modulus of shells was in the range of ~ 300–900 kPa, resembling the plant CWs of *Arabidopsis thaliana* shoot apical meristem and epidermal cells/dark grown hypocotyl [14]. Implementing 3D templating for CW-like material fabrication enables the investigation of more realistic CW properties, such as biomimetic permeability, mechanical stability, and deformation/expansion. Additionally, these CW-like materials respond to external stimuli, such as pH, humidity, and pressure in a biomimetic manner, providing insights into the dynamic behavior of plant CWs under varying conditions.

### In Silico Studies

**Fig. 4:**
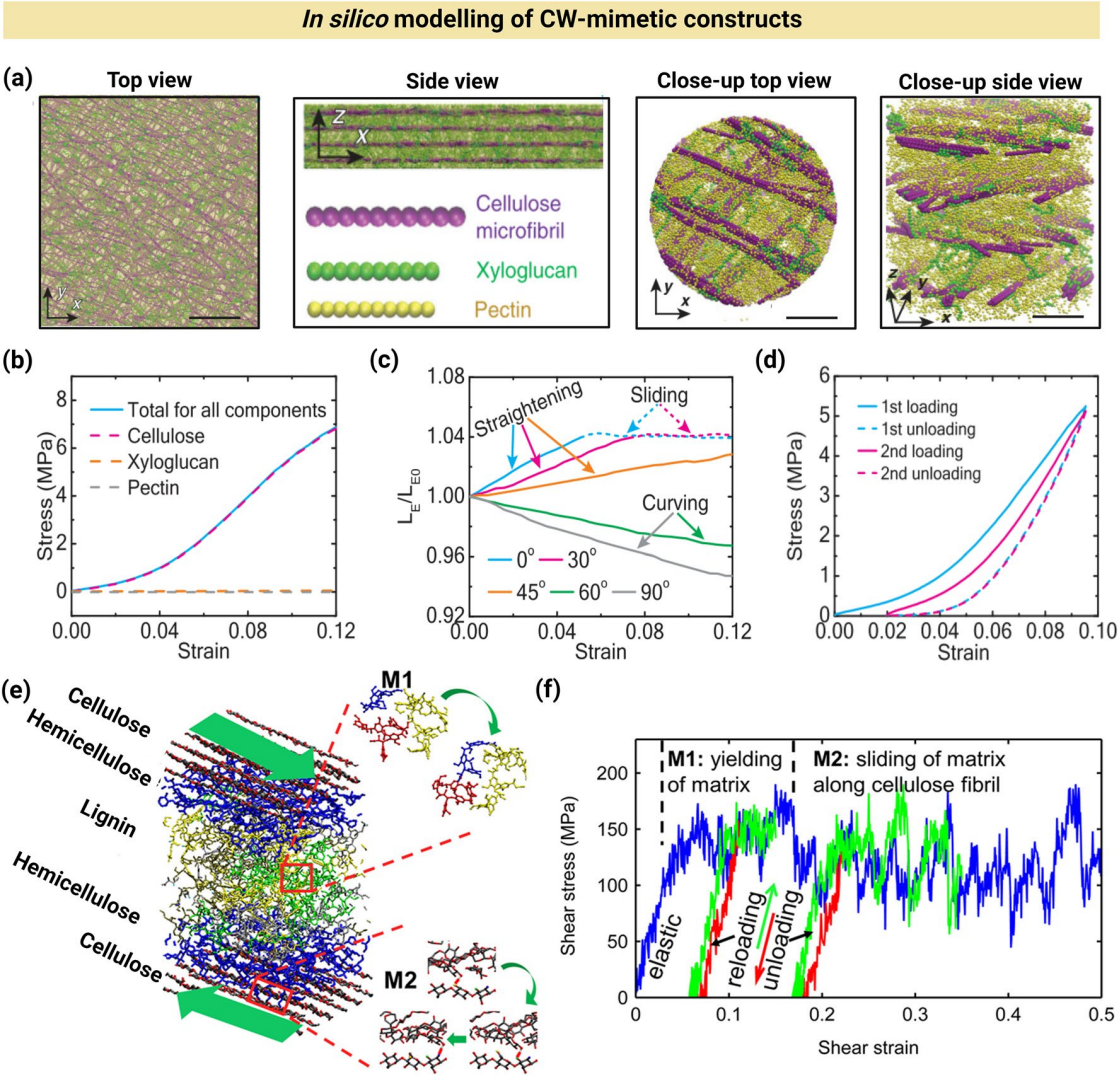
In silico studies of plant CWs using MD simulations.** a** Top and side views of a four-lamella CW after equilibration (Scale bars are 200 nm) along with their corresponding close-ups (Scale bars are 25 nm). Reproduced with permission [29]. Copyright Science, 2021. **b** Stress–strain behavior of PCW, consisting of CMF, xyloglucan, and pectin, showing that the stress is mainly tolerated by CMF. Reproduced with permission [29]. Copyright Science, 2021.** c** Normalized average end-to-end length of CMF (*L*_E_/*L*_E0,_ where *L*_E_ is the average end-to-end length and *L*_E0_ is its initial value at strain of 0%) as a function of strain, applied via the uniaxial stretching of single lamellae at varying CMF orientations of 0°, 30°, 45°, 60°, or 90°. Reproduced with permission [29]. Copyright Science, 2021.** d** Stress–strain response of onion epidermal CW during cyclic loading and unloading, showing a large hysteresis, stemming from energy dissipation and irreversible (plastic) deformation in the epidermal wall. Reproduced with permission [29]. Copyright Science, 2021.** e** Molecular model of wood CW, encompassing CNC, hemicellulose, and lignin molecules, assembled in a layered nanocomposite. M1 and M2 indicate the molecular orientation of CW matrix during yielding and sliding, respectively. Reproduced with permission [91]. Copyright Elsevier, 2015. **f** Stress–strain response in the CW model (blue), indicating 3 separate regimes: an initial elastic regime and two plastic regimes. The unloading (red) and reloading (green) curves indicate the irreversible deformation of CW after yielding. Reproduced with permission [91]. Copyright Elsevier, 2015

In silico studies of plant CWs using molecular dynamics (MD) simulations have been conducted to investigate how molecular interactions among CW components influence mechanical properties [15]. A comprehensive review on the in silico studies of plant biomass has recently been published, explaining the details of methodologies and mechanisms [89]. Here, we review several examples to illustrate the capabilities of MD simulations in studying plant CW analogs. Zhang et al., developed a coarse-grained MD model that offers insights into molecular mechanisms of PCW mechanics. The model consisted of chains of beads that represented the physical parameters of various wall polymers and their interaction potentials. After energy equilibration, it successfully yielded modeling results that accurately reflect the complex structure of CWs, where each lamella featured aligned CMF, embedded within matrix polymers (Fig. 4a) [29]. The model enabled the analysis of stress–strain responses of individual CW components, showing that CMF bore the stress, even at a high content of pectin and under the interactions of xyloglucans with CMF (Fig. 4b). This model also facilitated the mechanical analysis of single lamellae with varying CMF orientations, revealing distinct polymer movements and stress contributions that depended on orientation (Fig. 4c). Via cyclic loadings in the CW model, the mechanical mechanisms of energy dissipation in the wall were uncovered (Fig. 4d).

Overall, in silico models enable the study of CW structure–function relationships in ways that are not yet feasible experimentally. Coarse-grained models, in particular, simulate dynamic polymer behaviors that offer unique insight into the molecular mechanisms underlying macroscale mechanical behaviors of CWs. MD simulation has also been used to explore the structural, physical, and mechanical influences of hydration on wood CW. To construct a composite material mimicking softwood CW S2 layer via a bottom-up approach, CNC were modeled as a hexagonal packing of 36 chains each of which containing 10 monomer units [90]. CNC clusters were embedded in a composite matrix to mimic the CMF. Branched and unbranched lignin, GM, and xylan were modeled as multicomponent matrices with varying compositions to assimilate the distinct enriched domains surrounding the CMF. The CW S2 layer underwent highly anisotropic swelling with minimal swelling along the longitudinal direction and significant swelling in the transverse direction (170 times the longitudinal direction). The findings showed that the CW modulus and swelling along the longitudinal direction were mainly governed by CMF. Moreover, in contrast to CMF and lignin derivatives, hemicelluloses, interfacing lignin and cellulose, were ultrasensitive to water sorption. Therefore, water easily reached the cellulose-hemicellulose interfaces, disrupted the intermolecular hydrogen bonds, and weakened the fibril-matrix interface. The modeling results also indicated that the CW modulus and swelling along the transverse direction were mainly regulated by hemicelluloses. Lignin was a hydration-independent component, functioning as an interfibrillar space filler [90].

The mechanical behavior of wood CW under shear loading has been investigated using MD simulation to explore the underlying deformation mechanisms at the molecular scale. The shear loading was applied by pulling the outermost layers of cellulose in opposite directions. Figure 4e presents the molecular model of wood CW, encompassing cellulose, hemicellulose, and lignin [91]. The CW was modeled as a layered nanocomposite of stiff CMF and soft hemicellulose and lignin matrices. Figure 4f shows the stress–strain response of CW model with 3 separate regimes, including an initial elastic regime and two plastic regimes (i.e., the yielding of matrix and matrix sliding on the cellulose surface). By subjecting the composite to the shear loading, an elastic deformation followed by plastic deformation was observed. The plastic behavior was a result of matrix yielding (onset of plastic deformation) along the cellulose surface at shear strain of ~ 0.03, followed by sliding at shear strain of ~ 0.18. In nature, this type of “self-healing” interface is found in materials with excellent mechanical resilience [91]. In silico studies therefore enable the investigation of CW components at the molecular scale under stimuli, such as hydration, deformation/shear, and stretching/reorientation. Table 1 presents a summary of advantages and disadvantages of varying methods used for the micro-/nanoengineering of plant CW-like materials.

**Table 1:** Advantages and disadvantages of methods used for fabricating artificial plant CW-like materials

Fabrication method	Advantages	Disadvantages
Bacterial pellicle growth and impregnation	High purity and uniformity of bacterial cellulose [38] Mimicking biomechanical properties, such as extensibility and water sorption [10, 39, 40] Enabling the in vitro study of CW formation and interactions via bottom-up approaches [10, 39, 46, 47]	Structural differences with native plant CWs, as bacterial CMF have an Iα structure and are ribbon-like, while plant CMF (except algae) have an Iβ structure and are more like cylindrical wires [41–43] Randomly oriented cellulose ribbons [47], lacking the native CMF’s structural patterns [44, 45]
Layer-by-layer (LbL) assembly	Precisely controlling layer composition and thickness [59] Compatibility with varying substrates (e.g., charged, metal and biopolymeric surfaces) and multiple deposition methods (e.g., dip coating, spin coating, and pipetting) [59] Adaptability to different interactions (e.g., electrostatic, hydrogen bonding, and van der Waals forces) [61–63] Enabling multicomponent fabrication [64, 66]	Time-consuming and labor-intensive Complexity in thick layers especially for polymers with high viscosity or complex morphologies [62] Non-uniformity as a result of imbalanced charge density or polymer entanglement [65] Sensitivity to solvents (film thickness and uniformity are affected by solvents) [67]
Film casting	Scalable and biologically relevant [68, 69] Versatility in component inclusion [68, 70] Enabling the visualization of biomass recalcitrance [68, 69] Allowing physical and chemical crosslinking to tune mechanical and hygroscopic properties [70–74]	Time-consuming Heterogeneity due to the phase separation [69], leading to sample-to-sample variability Film properties are affected by humidity [77]
3D templating and particle coating	Geometrically relevant for mimicking 3D structures [78–81] Enabling the construction of multilayer shells [80, 83] Tunable permeability, mechanical stability, and elasticity, enabling the realistic studies of plant cell behavior [80, 83, 86] Enabling stimuli-responsive microcapsules, allowing for the investigation of plant CW-like dynamic behaviors [87]	Complexity in achieving consistent 3D structures [80, 83] Removal of templates or additional steps [80], increasing fabrication complexity and time Poor scalability [80, 87]
In silico	Uncovering molecular interactions and mechanisms that are difficult to assess experimentally [15] Simulating dynamic behaviors, such as stress–strain responses, hydration effects, and energy dissipation [29] Revealing CW structure–function relationships at the molecular level [90] Simulating anisotropic behaviors, such as the anisotropic swelling and mechanical responses of CWs at varying conditions (e.g., hydration and shear stress) [90] Facilitating bottom-up approaches for constructing composite models that mimic natural CWs and predict CW properties at multiple scales [90]	Significant computational resources and time [89] as well as dependence on the potential field used to model CW constituents Loss of accuracy in representing complex CW structures and behaviors as a result of model simplifications [29] Requiring experimental data for validation and calibration [91] Cannot fully replace experimental data, requiring real-world validation for most results [90] Limited to small systems due to computational constraints, which may not represent macroscopic behaviors accurately [90]

## Characterization Techniques to Assess CW-Like Materials

**Fig. 5:**
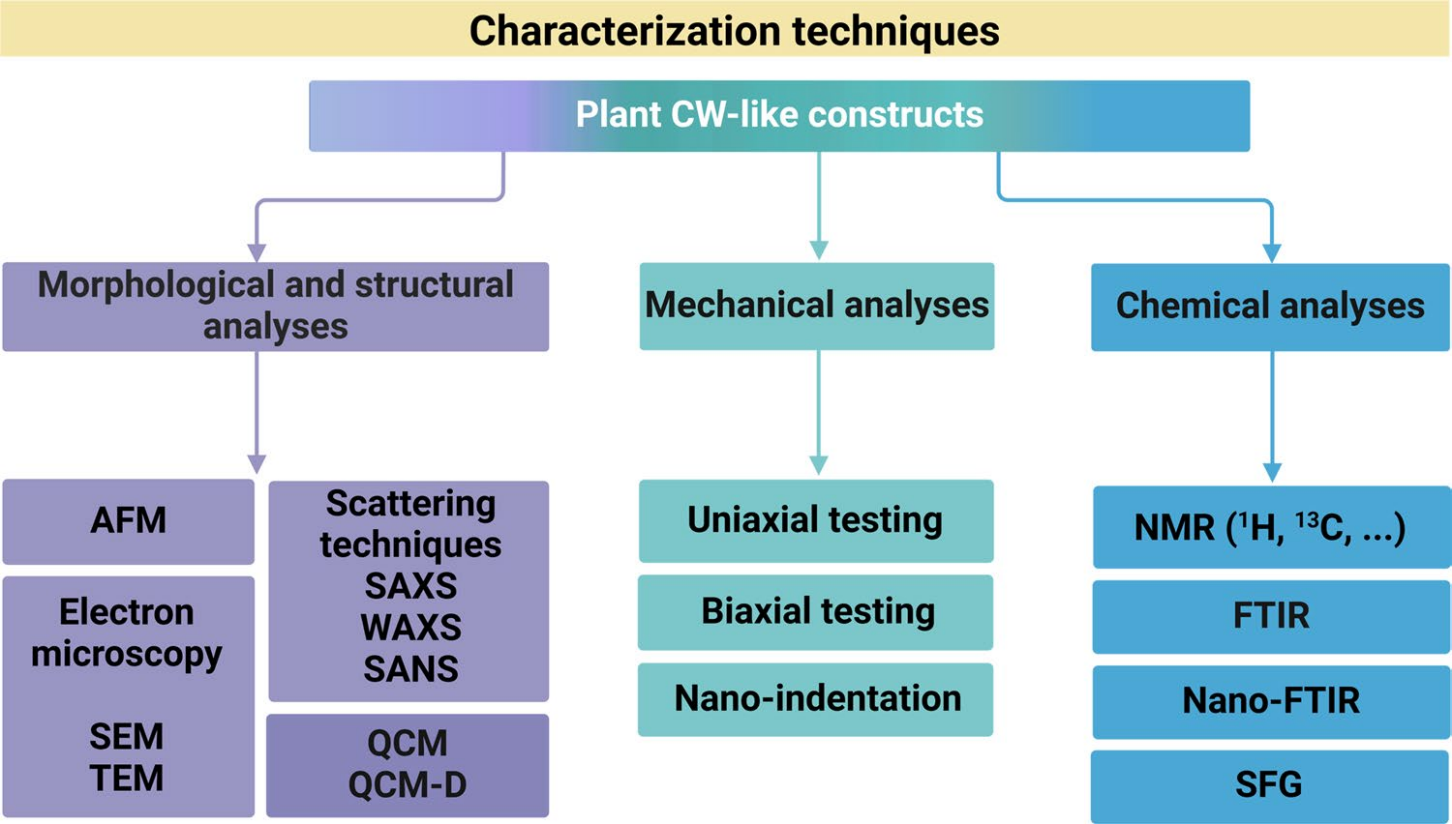
Varying techniques for mechanical, morphological, structural and chemical characterizations of natural and artificial plant CWs. Abbreviations are defined as follows: AFM (atomic force microscopy), SEM (scanning electron microscopy), TEM (transmission electron microscopy), SAXS (small angle X-ray scattering), WAXS (wide angle X-ray scattering), SANS (small angle neutron scattering), QCM (quartz crystal microbalance), QCM-D (quartz crystal microbalance with dissipation monitoring), FTIR (Fourier transform infrared), NMR (nuclear magnetic resonance), and SFG (sum frequency generation). Some abbreviations have been redefined here as per the request of a reviewer

Natural CWs and plant CW-like materials have been characterized from chemical, structural, and/or mechanical perspectives using varying techniques, summarized in Fig. 5. Here, we briefly describe these techniques and provide examples of analyses for both native and artificial CWs.

### Morphological and Structural Analyses

#### AFM

**Fig. 6:**
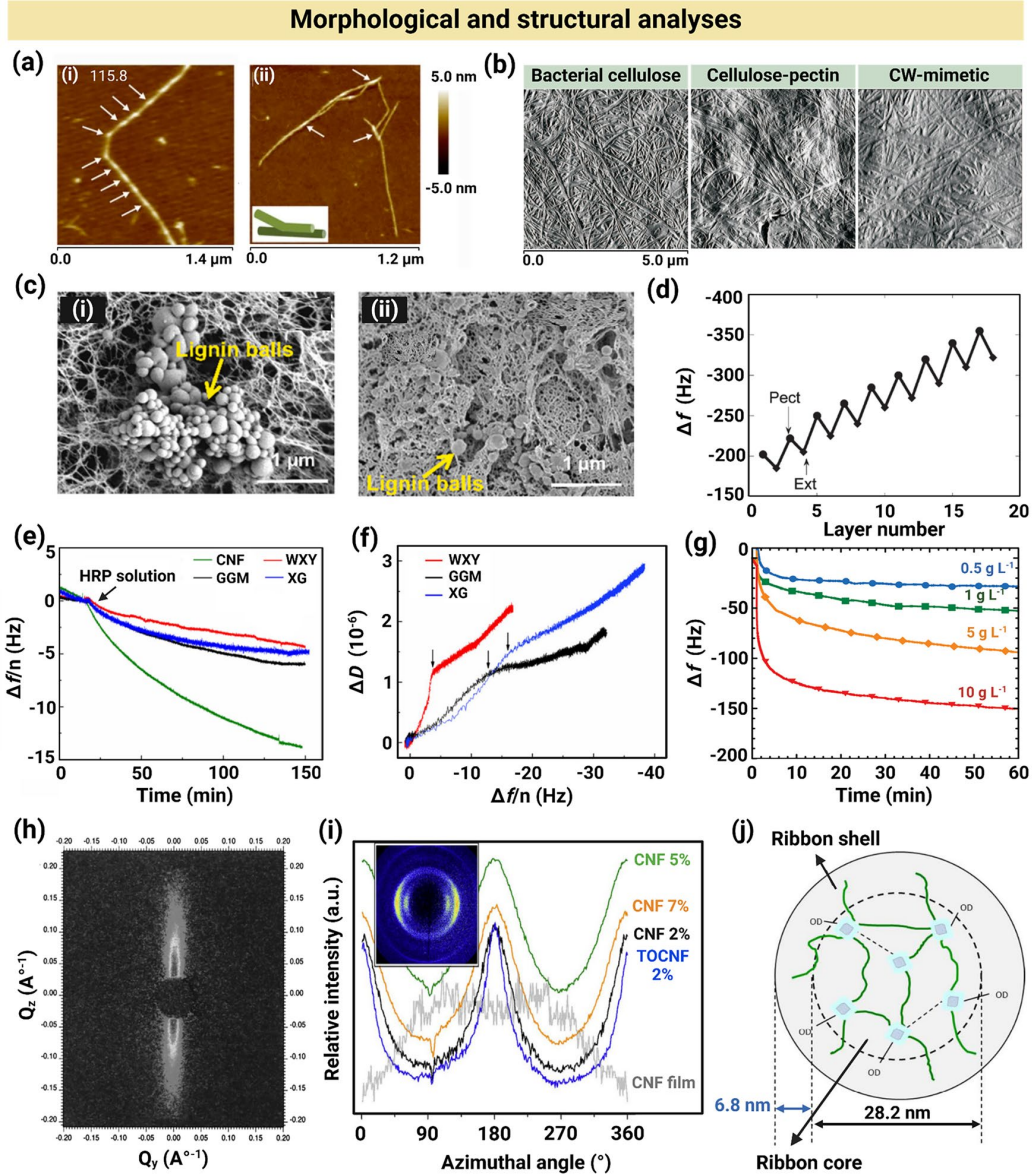
Morphological and structural characterizations of plant CW-like materials. **a** AFM height images of xyloglucan chains and xyloglucan assemblies. Reproduced under terms of the CC-BY license [95]. Copyright 2015, The Authors, published by Springer Nature. **b** AFM images of bacterial cellulose, bacterial cellulose-pectin, and bacterial cellulose-pectin-xyloglucan composites. Adapted under terms of the CC-BY license [96] Copyright 2010, The Authors, published by Institute of Agrophysics. **c** SEM images of physically and chemically crosslinked composites, consisting of cellulose/glucomannan/lignin, showing the extensive aggregation of lignin particles in physically crosslinked composites compared with the chemical analog. Reproduced with permission [73]. Copyright Elsevier, 2022. **d** QCM-based frequency changes (Δ*f*) versus deposited pectin (Pect) and extensin (Ext) layer number. Reproduced with permission [65]. Copyright Langmuir, 2010. **e** QCM-D-derived normalized frequency shift (Δ*f*/*n*) of hemicelluloses, including xyloglucan (XG), galactoglucomannan (GGM), and water-soluble xylan (WXY), initially deposited on bacterial cellulose films, showing the effect of HRP adsorption on polysaccharides over time. Reproduced with permission [54]. Copyright Springer Nature, 2021. **f** QCM-D-derived dissipation shift (Δ*D*) versus Δ*f*/*n* of hemicellulose adsorption to CNF. Reproduced with permission [54]. Copyright Springer Nature, 2021.** g** QCM-derived Δ*f* for xyloglucan adsorption to CNC-spin-coated quartz crystals versus time. Reproduced with permission [62]. Copyright Langmuir, 2010. **h** The 2D SAXS pattern of water-swollen horizontally-oriented individual single flax fiber. Reproduced with permission [106]. Copyright American Chemical Society, 1998. **i** Azimuthal integration of X-ray diffractograms in wet-spun filaments, obtained at a scattering vector of 15.8 nm^−1^. Reproduced under terms of the CC-BY license [112]. Copyright 2016, The Authors, published by Springer Nature. **j** A representative core–shell model, proposed for the structure of hydrated bacterial cellulose-xyloglucan composites. Reproduced with permission [119]. Copyright Royal Society of Chemistry, 2016

AFM is one of the most frequently used microscopy techniques for mapping the topography of intact plant CWs and CW-like composites at the nanoscale [92–94]. Compared with other microscopy techniques, AFM may be more advantageous under physiological conditions as it requires less sample preparation and typically avoids extensive drying processes. This is particularly important for studying CW-like materials that mimic the native, hydrated tissues [27]. Nanostructure and assembly of xyloglucan isolated from tamarind seed as a model system were investigated via AFM. Figure 6a shows AFM height images of self-assembled bundles of xyloglucan chains. The topological image (Fig. 6a-i) showed that xyloglucan had a rod-like morphology with length ~ 640 nm and height ~ 2.3 nm. Furthermore, an individual xyloglucan fibril had a helical structure with a period of ~ 115 nm and bending angle of ~ 128°. As observed in Fig. 6a-ii**,** xyloglucan chains were able to form parallel assemblies of fibers (shown with white arrows). The parallel assembly resembled the mechanism of xyloglucan linking to cellulose in PCWs, where a smooth, flat region of xyloglucan attached to cellulose through hydrogen bonds [95].

AFM has also been used to visualize and compare nanostructures in the native apple CW, reconstituted CW, and artificial CW-like composites. The artificial composite consisted of bacterial cellulose (26%), pectin (44%), and xyloglucan (20%), making up ~ 90% of dry polysaccharide mass in PCWs, and had similar morphological and structural features to native PCW models. Figure 6b shows the AFM images of artificial bacterial cellulose, bacterial cellulose-pectin, and bacterial cellulose-pectin-xyloglucan composites [96]. CMF in a bacterial cellulose-pectin-xyloglucan composite were thicker (~ 75 nm) compared with those in bacterial cellulose (~ 59 nm) and bacterial cellulose-pectin (~ 68 nm) composites. The thickness variations suggested that the bacterial CMF were coated with a monolayer of xyloglucan, consistent with PCW models. Composite roughness was determined by analyzing the AFM images and calculating the deviation of surface height values from the mean surface height over a given area. This deviation was then normalized by dividing it by the root mean square (RMS) roughness of surface. Therefore, the roughness value, a dimensionless quantity, indicated the degree of variation in the height of surface features. The roughness of artificial bacterial CWs (12) was comparable to that of natural CWs (13.1) [96]. Overall, AFM may combine nanoscale imaging with indentation-based mechanical measurements, providing unique insights into the structure–function of CWs and CW-like materials.

#### SEM and TEM

Electron microscopy techniques, such as SEM and TEM, have been extensively used to visualize the morphology and alignment of CMF and other CW biopolymers, such as hemicellulose, pectin, and lignin particles at the micro- and nanoscales [97, 98]. Deep-etch freeze-fracture TEM, which involves rapidly freezing the sample to prevent network disruption by ice crystal formation and producing clear, high-resolution replica images, was used to investigate the morphology of CMF in CW-like composites. This technique, applied to bacterial cellulose pellicles and xyloglucan composites, revealed a highly aligned, cross-bridged structure of CMF within the composites [46]. The association of xyloglucan with CMF enabled lateral alignment, which was not observed in the TEM image of xyloglucan-free composite (Fig. 6b). Thin strands of xyloglucan were detectable in the composite TEM image, which were distinguished as bridges (length ~ 20–70 nm), linking adjacent fibers and creating a ladder-like structure [96].

SEM was used to examine the surface coverage and integrity of hollow CW-like microcapsules formed via water–oil emulsification. Since emulsions could not be imaged using SEM, styrene particles were used to replace the oil cores. Styrene-water emulsions were prepared with the same CW-like composition and emulsification technique to facilitate imaging. SEM images showed a homogenous distribution of CNC (nanorods) parallel to the droplet surface, independent of CNC length; however, a dense interfacial network was observed for short nanorods (length ~ 189 nm) compared with long ones (length ~ 4000 nm). Loose networks of long nanorods limited dense packing as a result of steric hindrance and resulted in a porous multilayer organization of CNC [85].

SEM has also been used to image the internal structure of physically and chemically crosslinked CW-like cellulose/GM/lignin composites. SEM images showed that the pore of both kinds of composites became finer and denser by adding lignin (5–20 wt%). As presented in Fig. 6c-i, highly aggregated lignin balls were formed in the physical composites, whereas no apparent aggregation of lignin nanoparticles occurred in the epichlorohydrin-mediated chemically crosslinked composites (Fig. 6c-ii), corresponding to the formation of covalent ether bonds between lignin-cellulose or lignin-GM [73].

#### QCM and QCM with Dissipation Monitoring (QCM-D)

QCM is a surface-sensitive, real-time tool to determine the thickness of deposited layers in CW-mimetic materials, wherein polysaccharides adsorb to substrates such as films and beads. When a species is adsorbed to the QCM sensor surface, the increase in mass at the nanogram to microgram levels is correlated with the changes in the resonance frequency of a quartz crystal resonator. The Sauerbrey equation is used to calculate the mass of adsorbed layers when the added layers are thin, rigid, and firmly attached to the sensor surface [64, 99, 100]. QCM has been used to evaluate the LbL growth of pectin-extensin films [65]. Figure 6d presents the frequency shift of a QCM crystal (Δ*f*) versus the number of deposited pectin and extensin layers. When pectin was deposited, Δ*f* decreased, indicating successful pectin adsorption; however, upon extensin deposition, Δ*f* increased, which was attributed to extensin low charge density (with only ~ 5% of monomers carrying charge). This resulted in material removal from the surface due to weak electrostatic interactions. The Δ*f* of a total sorption cycle (pectin-extension adsorption) decreased, indicating LbL film growth [65].

QCM-D, an extended version of QCM technique, has been used to study the interactions between cellulose, lignin, and hemicelluloses, including xylan, galactoglucomannan, and xyloglucan [54]. QCM-D measures not only Δ*f* but also a parameter related to energy loss or dissipation, known as dissipation shift (Δ*D*). While Δ*f* reflects changes in the mass attached to the sensor surface, Δ*D* provides information about the viscoelastic properties of adsorbed layer. QCM-D is used to characterize mass deposits, which induce frictional dissipative losses because of their viscoelastic nature. Higher Δ*D* values indicate that the material is more viscous or energy-absorbing, whereas lower Δ*D* values suggest that the material is more elastic or energy-preserving [101]. Accordingly, QCM-D was used to determine the adsorption of hemicelluloses and lignin in the form of DHP on CNF to investigate the effect of cellulose, xylan, galactomannan, and xyloglucan on lignification. Figure 6e shows the normalized Δ*f* profile during HRP adsorption to polysaccharide matrices. To study lignification, QCM sensors were spin-coated with thick layers of CNF (> 4000 ng m^−2^) and either used uncoated or coated with a thin layer of hemicellulose. HRP was then deposited onto these surfaces. The value of Δ*f*/n (*n* denotes the overtone number) for the sensor coated with only CNF was the lowest (-15 Hz after 150 min) at all deposition time points and decreased most rapidly, indicating a higher amount of HRP adsorption and a faster adsorption rate on CNF-coated sensors compared with the hemicellulose-coated CNF, which had a Δ*f*/n of ~ -5 Hz after 150 min. This increase in HRP adsorption to CNF was attributed to the hydrophobic interactions between CNF and HRP, which decreased by coating CNF with hemicelluloses. Figure 6f presents the Δ*D* of CNF-coated crystals versus Δ*f/*n upon hemicellulose adsorption to CNF surface. The Δ*D* values for xylan, galactoglucomannan, and xyloglucan rapidly increased (from 0 to 1 × 10^–6^) during the early stages of adsorption, implying the formation of a hydrated soft surface. This was followed by a gradual increase in Δ*D* as the adsorption layer became thick and difficult to harden [54].

QCM-D has also been used in situ to examine the kinetics of polysaccharide adsorption to CW-mimetic bacterial cellulose-based films [40] and to the interfaces of droplets within emulsions [87]. Xyloglucan adsorption to CNC-spin-coated quartz crystals over time was monitored using QCM, as shown in Fig. 6g. As the xyloglucan concentration increased from 0.5 to 10 g L^−1^, Δ*f* decreased, with a significant drop in Δ*f* observed within the first 5 min for all concentrations, indicating rapid adsorption during the LbL buildup of PCW-like CNC-xyloglucan films. Subsequently, a slight decrease in Δ*f* was recorded up to 60 min without reaching a plateau, which was attributed to the continuous self-rearrangement of xyloglucan chains on the surface, impairing the adsorption equilibrium [62]. In the fabrication process of PCW-inspired plantosomes, the QCM-D measurements showed a significant decrease in Δ*f* to -90 Hz and an increase in Δ*D* to 20 × 10^6^ after pectin adsorption to cationic CNF-coated crystals. In contrast, direct pectin adsorption to the crystal resulted in a decrease in Δ*f* and an increase in Δ*D*. These Δ*f* and Δ*D* values confirmed the formation of outer LbL CNF-pectin shell of plantosomes [87]. Combining QCM and surface plasmon resonance (SPR) can provide a more comprehensive understanding of interactions at interfaces, particularly in LbL assemblies. While QCM detects changes in the mass of adsorbed layer, including dry mass and any associated water, SPR measures changes in the refractive index of materials near the sensor surface, which occur when substances attach to or detach from the surface [102].

#### Scattering Techniques

Scattering techniques involve the use of radiation sources, such as X-rays or neutrons, to detect patterns scattered by the electrons or nuclei, respectively. For CW-like materials, scattering techniques provide information on the size, orientation, and/or arrangement of components that may not necessarily have a crystalline order [103, 104]. Small-angle X-ray scattering (SAXS) probes nanoscale features [103], and wide-angle X-ray scattering (WAXS) is more suited for crystallographic studies and molecular scale characteristics [105]. SAXS was used to investigate the arrangement of CMF in native flax CWs, without requiring additional treatments often necessary for other techniques like electron microscopy. For a horizontally oriented fiber, the scattering pattern appeared in the vertical direction, as shown in Fig. 6h [106]. The SAXS pattern of CMF in a single flax fiber also showed well-defined streaks, which was attributed to the semi-crystalline structure of microfibrils and void spaces among microfibrils [106, 107]. Indeed, the anisotropic SAXS pattern from CMF is well known; the fluctuations in scattering density in the equatorial plane (on a 10 Å length scale) arise from the electron density differences between the crystalline CMF and the void spaces, i.e., regions of less dense material.

SAXS has been used to investigate CMF orientation in native primary and secondary CWs as well as in synthetic CW-like materials [103]. For investigations on the SCW, flax fibers were studied because of their composition and thick SCW, rendering them a suitable representative for SCW. SAXS was used to study the hydration effect on the microstructure of flax fibers, and a considerable structural change was observed for flax fibers in the wet and dry states, demonstrated by the scattering patterns. The azimuthal intensity of CMF followed a Gaussian distribution, and the full width at half maximum (FWHM) decreased from 15° to 11° as the flax fibers transitioned from the wet to dry state. As water evaporated from the fibers and the scattering from water diminished, a meridional reflection appeared at a scattering vector of 0.095 Å^−1^. Upon fully drying, the meridional scattering intensity increased at a scattering vector of less than 0.06 Å^−1^, which indicated an increase in CMF alignment [108]. Although X-ray scattering investigations of SCW are more common than those of PCW because of the disordered cellulose packing and lack of fibril orientation, SAXS has successfully been used to determine the CMF orientation in PCW [109]. The changes in CMF orientation in native collenchyma CWs through various developmental stages were studied with SAXS. In the early growth stage, the SAXS pattern was isotropic, and upon further plant growth, it became anisotropic and vertically elongated, which suggested CMF alignment in a specific direction. Accordingly, the intensity profile of CMF initially exhibited a broad angular distribution (from 0° to 80°). As the development of CMF in the walls of collenchyma cells progressed, this distribution narrowed, eventually shifting to a range between 0° and 15° in the later stages of growth [110]. Using SAXS, the PCW structure of *Chara corallina* and *Arabidopsis thaliana* in the native hydrated state was analyzed, showing a bimodal microfibril angle distribution and indicating that the majority of CMF were oriented either longitudinally or transversely. These distributions had a broad scattering around mean microfibril angles of ~ 0° (longitudinally oriented microfibrils) and 90° (transversely oriented microfibrils).

WAXS has been used to determine the crystal type, crystallinity (%), and cellulose alignment and orientation within hydrated CW-like composites [103, 111]. WAXS diffractograms of wet-spun filaments, made by spinning CNF hydrogels or colloidal suspensions with solid contents typically between 2% and 7%, had the characteristic peaks of cellulose I crystals at a scattering vector of 15.8 nm^−1^. This indicated that the wet-spinning process did not affect CNF crystallinity. The degree of orientation *f*_*c*_ = (180 – FWHM/180) × 100 was calculated by extracting the FWHM from the azimuthal intensity distribution profile. Figure 6i presents the azimuthal integration of X-ray diffractograms, showing fibril orientations in wet-spun filaments, recorded at a scattering vector of 15.8 nm^−1^. Filaments made up of TEMPO-oxidized CNF (2%) had the highest degree of orientation (~ 83%), tensile strength (297 MPa), and Young’s modulus (21 GPa) [112]. WAXS has shown that CNF, produced via dry spinning, may be aligned within single-filament fibers, which is reflected in equatorial arc patterns in the diffractograms. The arcs were identified as the $1\overline{1}$ 0 and 200 cellulose planes, which confirmed the alignment of CNF. The degree of orientation had a maximum value of 0.68 for the filaments spun at a high spinning rate (216 mm s^−1^) because of a high external shear force [113].

WAXS has also been used to study the alignment of bacterial CNF that were assembled into macrofibers via wet-spinning and stretching. These macrofibers mimicked the mechanical properties of native cellulose bundles, with tensile strength of 6–7 GPa and Young’s modulus of 120–140 GPa. The WAXS patterns that were obtained from an untreated bacterial CNF film showed reflections corresponding to the (200) and ($1\overline{1}0$) planes, which indicated the random orientation of untreated nanofibers; however, the WAXS pattern of wet-spun aligned macrofibers had an arc pattern of ($1\overline{1}0$) and (200) reflections, implying crystallite alignment along the longitudinal axis of fibers. As the stretching ratio (SR) increased, the more defined reflections and narrower FWHM in the scattering patterns confirmed a greater degree of alignment in the macrofibers [114]. In addition, WAXS can detect changes in the length of crystalline cellulose via the peak position of (004), which corresponds to a quarter of the unit length of cellulose. This allows for the analysis of the nanoscale load-bearing properties of cellulose under tension. Studies have demonstrated that cellulose can withstand significant stress, exceeding 600 MPa, in PCW and SCW [115]. Further research on this characteristic is warranted to provide deeper insights into how nanoscale polymer deformations contribute to the overall mesoscale load-bearing abilities of CWs.

SANS has been conducted to examine hydrogen bonding patterns in cellulose Iβ. This technique was used to characterize films, prepared via aligning tunicate-derived CNC [116]. The hydrogen atoms participating in the intramolecular O3…O5 hydrogen bonds yielded spherical and well-defined positions in SANS diffraction patterns. In contrast, the hydrogen atoms on O2 and O6 appeared as non-spherical density peaks and were split across multiple locations (i.e., not well-defined), indicating poorly defined positions. This suggests the presence of multiple geometries with varying hydrogen atom arrangements. Neutron refinement, the process of obtaining agreement between experimental scattering data and the structural model, suggests a more complex and disordered hydrogen-bonding network for cellulose Iβ films compared with a single-crystal structure. This observation was demonstrated by the *R*-factor (also called the residual factor), which is a parameter describing how well the model and data agree. The *R*-factor was fairly high for cellulose Iβ films (0.2095) compared with the typical values for a single crystal structure (e.g., *R*-factor of 0.085 for β-D-cellotetraose) [117]. The neutron scattering length of hydrogen is negative (− 0.37 × 10^−12^ cm), whereas that of deuterium is positive (0.667 × 10^−12^ cm) [118]. Consequently, components of interest for SANS analysis can be deuterated (i.e., replacing hydrogen atoms with deuterium) to render the hydrogen bonds identifiable in SANS experiments.

To investigate the role of CW polysaccharides in the cellulose biosynthesis process, native bacterial cellulose and CW-like bacterial cellulose composites containing arabinoxylan (53%) and xyloglucan (27%) were characterized using SANS [119]. As opposed to the previous studies describing bacterial cellulose ribbons as a one-phase solid material, the SANS profile was well-fitted to a core–shell model. Figure 6j shows a representative core–shell model proposed for bacterial cellulose-xyloglucan ribbons, encompassing a core of impermeable cellulose crystallites and a shell of partially hydrated paracrystalline cellulose networks. The neutron scattering length density (SLD) of core (1.87 × 10^–10^ cm^−2^) was different from the shell (< 3.66 × 10^–10^ cm^−2^). The SLD discrepancies indicated that the strong hydrogen-bonded networks holding CMF together impaired the solvent accessibility to the core regions of ribbons. This resulted in varying degrees of solvent exchange, with about 60% solvent exchange within the core for pure cellulose and approximately 68% for the bacterial cellulose-xyloglucan composite. Furthermore, fitting-derived parameters indicated that both xyloglucan and arabinoxylan were deposited on the surface of bacterial cellulose ribbons via non-specific adsorption mechanisms. Such deposition provided additional hydroxyl groups in the shell of ribbons, facilitating solvent access to the core. Altogether, SANS analysis implied that only xyloglucan established strong interactions with the CMF of core region via crystallization/assembly [119]. The xyloglucan existence within the core region plays crucial roles in decreasing the total crystallinity, creating plant-characteristic Iβ allomorph and lowering the packing density by individualizing CMF [119, 120].

### Mechanical Analyses

#### Uniaxial and Biaxial Mechanical Testing

For assessing the mechanical properties of CW-like films and bacterial pellicles, uniaxial and biaxial mechanical tests have been used. Uniaxial tensile testing provides characteristics such as tensile strength, Young’s modulus, work of fracture, and strain at fracture [10, 51, 53]. Similarly, uniaxial compression and flexural testing provide quantitative information about the strength, stiffness, and elasticity of films at different modes of deformation. Uniaxial tensile set-up may also be rationally modified to mimic the effects of in planta phenomena, including turgor pressure, creep, stress-relaxation, and extension, on CW-like matrices [39, 51, 121].

**Fig. 7:**
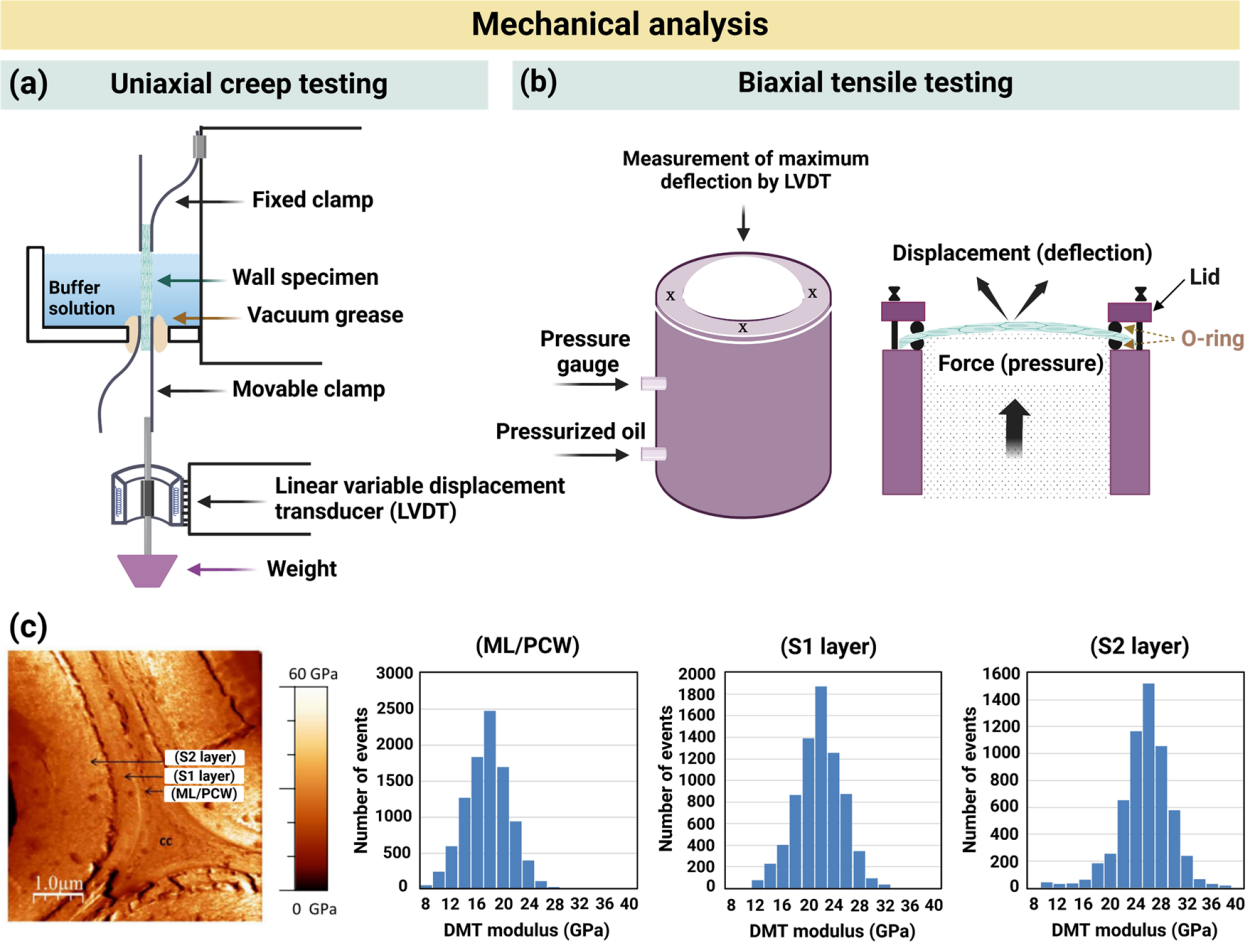
Mechanical characterizations of plant CW-like materials. **a** Uniaxial creep test setup to measure the time-dependent displacement of viscoelastic CW-like materials at a constant tension. Adapted with permission [121]. Copyright Springer Nature, 1989. **b** A customized and unique biaxial tensile testing setup was used to apply pressure to films and hydrogels from one side while measuring the corresponding deflections, providing insights into their mechanical responses. Adapted with permission [51]. Copyright Springer Nature, 2002. **c** Topological image and indentation modulus of poplar fiber CW layers, obtained using AFM, which show three distinct regions, ML/PCW, S1 layer, and S2 layer. Cell corner is denoted as cc. Reproduced under terms of the CC-BY license [69]. Copyright 2017, The Authors, published by Springer Nature

Accordingly, creep is often measured by customized uniaxial constant-force extensometers where the top clamp is fixed, and the lower movable clamp applies force (e.g., 20-g force). Figure 7a shows the scheme of a uniaxial constant-force extensometer, registering the time-dependent displacement of viscoelastic CW-like materials at a constant tension [121]. The long-term extension of cucumber hypocotyl CW, analyzed by the extensometer, showed that the creep behavior depended more on the activities of enzymes attached to or entangled in the CW, rather than the wall viscoelastic properties. Subjecting CW to denaturants, high temperature, very low or high pH, or metal cations inhibited the creep activity, likely due to the enzyme deactivation. However, these treatments, except pH and copper ion (Cu^2+^) exposure, had no apparent effect on the CW viscoelastic behavior [121].

The uniaxial extensometer testing was also conducted on impregnated bacterial cellulose/hemicellulose composites, containing an expansin (CsExp1), to investigate molecular targets for the expansin in PCW. The expansin increased the extension of xyloglucan composites by ~ 75%, whereas it had no effect on GM or galactomannan composites. The expansin also enhanced the extension of cellulose-only materials, although this increase was not detectable in a constant-load extension test as a result of inherent high material stiffness and insufficient external force [39]. Although the extensometer setup is straightforward and cost-effective, the heterogeneity of cell tissues poses a challenge in determining the precise contributions of CW components to the mechanics of assembled plant tissues. This complexity arises from the interplay of tissue geometry, turgor pressure, and water flux [121].

A bulge-testing device has also been customized to perform biaxial tensile testing on highly hydrated CW-like composites, enabling the mimicry and analysis of biaxial deformations occurring during CW expansion. Figure 7b presents a scheme of biaxial tensile testing setup by which pressure is applied to plant CW-mimetic films or hydrogels from one side, and the corresponding deflection is registered. Given the cell's spherical shape, the force exerted on the CW by the cell components and cytoplasmic membrane leads to uniform biaxial cell stretching. Films were secured to the orifice with O-rings and a lid, and deflection or pressure was measured using a linear variable displacement transducer (LVDT). According to the pressure–displacement curves, a substantial pressure (⪆ 400 kPa, may vary depending on the device geometry) was required to deform bacterial cellulose films, which indicated that the films had high stiffness and strength, as measured by this specialized biaxial tensile testing setup [51].

#### AFM Nano-Indentation

AFM may be used for imaging (contact, tapping, or non-contact modes) or mechanical property measurement (force-distance mode) [122, 123]. AFM has been used to evaluate the mechanical properties of microscopic areas using the force-distance mode (e.g., nano-indentation), rendering it ideal for analyzing layers within plant tissues or CW-like materials [69, 86]. Nano-indentation enables measuring force versus displacement at a high resolution and small scales, furnishing mechanical properties, such as elastic modulus and hardness. To calculate the modulus from the force-distance curve, an appropriate contact model must be tailored to the specific contact geometry between the material and the cantilever tip. Hertz, Johnson-Kendall-Roberts (JKR), and Derjaguin-Muller-Toporov (DMT) are common models used for flat and stiff films in varying conditions [69, 123, 124]. The Hertz model is commonly used for the contact between two elastic solids (e.g., flat and stiff films) under the assumption of small deformations and negligible adhesion forces. Therefore, this model fails to consider the viscoelastic properties of biological samples and is invalid at large deformations even for linear elastic materials [125, 126]. The JKR model is used for considering the contact between compliant materials with non-negligible adhesion forces. It accounts for the deformation of contacting surfaces and their corresponding adhesive forces. The JKR model is used to analyze the contact behavior of soft films or films with significant adhesion [127]. The DMT model is used for analyzing the contact between hard elastic materials with adhesive forces [126, 128, 129], which considers the adhesion between two surfaces [126]. The choice of model depends on the specific characteristics of composites, including stiffness, deformability, and adhesion.

For SCWs, AFM can measure the nanoscale indentation modulus and image detailed features of CMF, revealing structural aspects such as microfibril angles. These details are crucial for understanding how the CWs resist compression and provide mechanical support. These properties can be readily characterized for SCW materials such as wood, which are accessible and simple to prepare [130]. Muraille et al., probed the stiffness of poplar stem, a type of hardwood, and artificial SCW films using the AFM nano-indentation and DMT model. Due to the sharpness of AFM tips, the DMT model was used. Figure 7c presents the AFM image and indentation modulus of poplar fiber CW layers. AFM topological images and distinct moduli of CW layers confirmed three layers, including the ML/PCW (~ 17 GPa) and two SCW layers (S1 ~ 21 GPa and S2 ~ 26 GPa) [69]. In comparison, SCWs from softwood such as spruce stem are generally softer (~ 15 GPa) [131]. In contrast, CWs from certain crop stalks can reach indentation moduli of 16–20 GPa [132]. The indentation modulus of CW-mimetic ternary composites was 11 and 22 GPa for CNC/GM/DHP and CNC/xylan/DHP, respectively, agreeing with the values observed for the CW of poplar fibers [69].

AFM is also used to characterize the out-of-plane indentation mechanics of PCWs for two primary reasons. First, PCW samples are generally too soft and thin to resist in-plane compression for meaningful AFM measurements. Second, AFM nano-indentation can apply piconewton forces, inducing only nanometer-scale indentation in micron-thick PCWs, allowing for accurate measurement of the out-of-plane indentation modulus [49]. In some cases where isolating PCW for tensile testing is difficult, AFM is used to probe the nanoscale in-plane wall mechanics in their native state, assuming that the indentation is deep enough to involve the in-plane wall extension [49]. Interpreting data from living cell or tissue measurements requires caution, as factors such as turgor pressure and complex cell geometry can significantly influence the results [133]. In this context, the onion epidermal wall serves as a valuable model system for studying out-of-plane indentation mechanics, which are distinct from tensile mechanics and are influenced by matrix polymers, such as pectin.

### Chemical Analyses

#### NMR Spectroscopy

NMR spectroscopy provides valuable insights into the molecular structure and dynamics of native and artificial CWs via elucidating the local chemical environment of specific nuclei [134, 135]. NMR is sensitive to isotopes with non-zero spins, such as ^13^C and ^1^H in solid or liquid states. Cross polarization/magic angle spinning (CP/MAS) solid-state NMR (SS-NMR) spectroscopy has been conducted to investigate the interactions of cellulose-hemicellulose-lignin components [46, 47] and to quantify the crystallinity of native CWs and CW-mimetic composites with enhanced signal resolution compared with traditional SS-NMR [47, 120]. The crystallinity of cellulose within the composites was determined by calculating the ratio of the crystalline C4 peak area, found in the 85–92 ppm chemical shift region, to the total peak area in the 80–92 ppm region. The non-crystalline content of bacterial cellulose grown in a xyloglucan solution was 47%, suggesting that xyloglucan significantly decreased the cellulose crystallinity, which was initially 80%-85% [47].

The solid state ^13^C NMR analysis also showed that composites encompassing cellulose and 80%-85% rigid xyloglucan segments were likely aligned with cellulose chains, which made the chemical shifts, particularly glucosyl C-1, different from the chemical shifts of the flexible portion of xyloglucan. Only a small fraction of xyloglucan in the composite performed as cross-bridges [46]. The NMR spectra of cellulose/GM composites showed that the mannan segments bind to cellulose, followed by the conformational transition to an extended form. Through resolution enhancement, additional peaks emerged between 102–103 ppm, attributed to the C-1 atoms of mannan structural units adopting an extended "cellulosic" conformation, a pattern also observed in crystalline mannan. Integration of C-4 crystalline and non-crystalline signals implied a remarkable reduction in cellulose crystallinity from 80%-85% to 25% [46]. Note that crystallinity values obtained from different techniques should not be directly compared, as each method is based on distinct physical principles and may detect different facets of crystalline order [136].

**Fig. 8:**
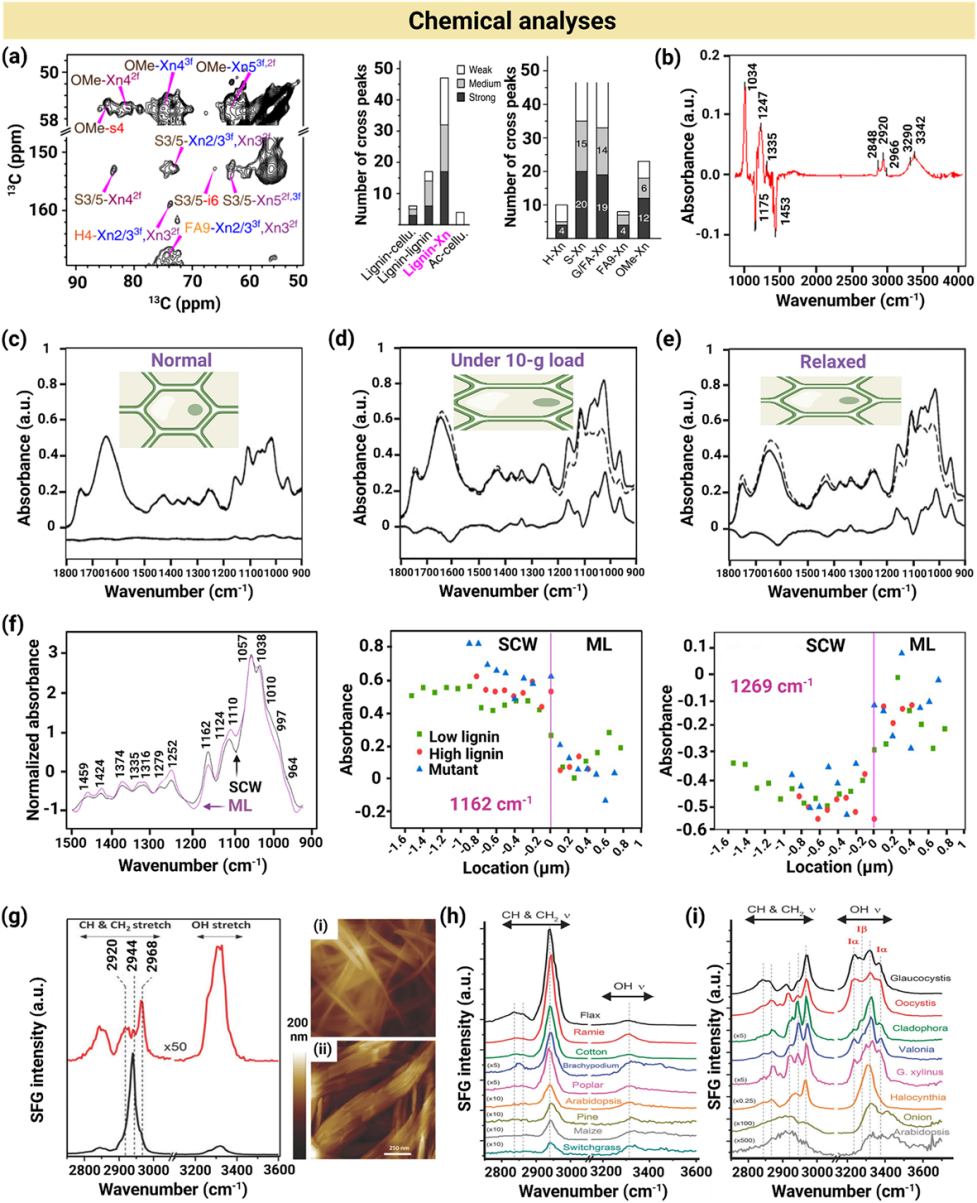
Chemical characterizations of plant CWs and CW-like materials. **a** A representative 2D ^13^C NMR spectrum of maize lignin (represented by S, G, H, and FA labels) and cellulose (i: interior, s: surface), showing cross-peaks that represent interactions between the components. S, G, H, and FA are maize lignin residues. Xn1-4 (2f, 3f), OMe, and cellu. stand for C1-C4 of xylan (twofold and threefold conformations, respectively), lignin methoxy group, and cellulose, respectively. The spectrum provides information on the quantity of cross-peaks between lignin and polysaccharides or lignin and xylan. Reproduced under terms of the CC-BY license [37]. Copyright 2019, The Authors, published by Springer Nature. **b** FTIR spectrum of CNC-polyethyleneimine microcapsules, showing the peak associated with vibrational OH stretching at 3342 cm^−1^, which typically appears at 3500 cm^−1^. Reproduced with permission [140]. Copyright American Chemical Society, 2015. FTIR microspectroscopy spectra of **c** initial, **d** stressed, or **e** relaxed plant cells, including parallel (positive, solid line) and perpendicular (positive, dashed line) polarization and subtraction (mainly negative, solid line) spectra. Reproduced with permission [143]. Copyright Oxford University Press, 2000. **f** Nano-FTIR spectra of SCW and ML of poplar fibers along with Nano-FTIR absorbance versus location of AFM tip for wavenumbers pertaining to polysaccharides (1162 cm^−1^) and lignin (1269 cm^−1^). Absorbance normalization was conducted via standard normal variate (SNV) transformation method: [(absorbance – average absorbance from all wavenumbers) / absorbance standard deviation]). Reproduced with permission [124]. Copyright American Chemical Society, 2022.** g** SFG spectrum of randomly-packed (red) and uniaxially-aligned (black) cellulose Iβ crystals with corresponding AFM images (i) and (ii), respectively. Reproduced with permission [148]. Copyright Royal Society of Chemistry, 2014. **h** SFG spectra of the SCW of several land plants, including flax, ramie, cotton, *Brachypodium*, poplar, *Arabidopsis*, pine, maize, and switchgrass. Reproduced with permission [148]. Copyright Royal Society of Chemistry, 2014. **i** SFG spectra of biological tissues, including algal CWs (*Glaucocystis*, *Oocystis*, *Valonia*, and *Cladophora*), cellulose biofilm produced from *G. xylinus*, *Halocynthia* mantle, onion epidermis, and *Arabadopsis* aerial tissue [148], showing that the relative intensity of the hydroxyl to alkyl signals was significantly greater for these PCWs (as shown in panel **i**) compared with most SCWs (panel **h**). The higher intensity suggested that PCWs had a lower degree of antiparallel CMF orientation over the SFG coherence length scale. Reproduced with permission [148]. Copyright Royal Society of Chemistry, 2014

SCWs of model plants *Arabidopsis*, maize, switchgrass, and rice were analyzed using SS-NMR to reveal lignin-polysaccharide interactions and the effect of hydration on CWs [37]. The plants were cultivated in a closed chamber of ^13^CO_2_, enabling ^13^C labeling. This isotopic enrichment provided 2D carbon spectra with exceptional sensitivity and atomic resolution, facilitating the investigation of site-specific hydration and lignin-polysaccharide interactions in their native state. Cross peaks arising from a sub-nanometer contact between two different atoms in the neighboring molecules provided the details of CW polymer spatial proximities. The intact maize stems had 74 intermolecular cross-peaks in a long-mixing (1.0 s) 2D spectrum. As a result, lignin was found to predominantly bind xylan (Xn), not cellulose. The interaction between cellulose and lignin was minor, challenging the previously held belief that lignin acts as a glue, binding CMF and hemicellulose together [137, 138]. Note that maize lignin mainly consists of syringyl (S), p-hydroxyphenyl (H), guaiacyl (G), and ferulate (FA) residues. Binding lignin to xylan depended on physical contact, particularly electrostatic interactions. This was based on the direct correlation between the number of physical contacts with polysaccharides and the number of methyl ether groups (OMe) in lignin. Figure 8a shows a representative ^13^C NMR spectrum of lignin-xylan-cellulose cross-peaks and the quantity of cross-peaks of lignin-polysaccharides and lignin constituent units-xylan. Accordingly, 80% of the OMe-Xn cross-peaks were strong or medium, implying the electrostatic-mediated physical contact between lignin methoxy groups and Xn polar entities. Additionally, “water-edited” correlation experiments were conducted to study the site-specific hydration [139]. Water-edited ^1^H-^13^C spectra of SCWs suggested weaker water associations compared with PCWs, confirmed by 2–4 times slower ^1^H polarization transfer. Within just 4 ms of ^1^H mixing time, lignin and polysaccharide peaks in maize SCW reached only 20%-30% of their equilibrium intensity. This is notably lower than the 60%-80% for pectin and 30%-40% for cellulose observed in *Arabidopsis* PCWs, which was attributed to the hydrophobic lignin, tighter polymer packing, and the lack of pectin with its water-stabilizing/binding property [37].

#### FTIR Spectroscopy and Microspectroscopy

FTIR is a versatile tool to analyze a chemical vibrational response to the infrared (IR) radiation, exposing the fingerprints of functional groups within a material. The vibrational modes may shift depending on component interactions, which can provide information on chemical functionality, molecular conformation, crystallinity, and hydrogen bonding [9, 128, 129]. FTIR spectroscopy has been used to probe native plant tissues and artificial CWs in biologically relevant environments and in situ processes. Accordingly, the FTIR analysis of artificial sphere-like CWs, comprising bacterial cellulose, pectin, and hemicelluloses, elucidated changes in cellulose allomorph, crystallization, and hydrogen bonding in the presence of pectin and hemicelluloses [68, 70, 111, 140]. Incorporation of xylan and xyloglucan into bacterial cellulose culture media led to a shift in the cellulose allomorph from Iα, typically associated with bacterial sources (characterized by wavenumbers ~ 3240 and 750 cm^−1^), to Iβ, which is commonly found in higher plants (indicated by wavenumbers ~ 3270 and 710 cm^−1^). The FTIR analysis of cellulose-xylan and cellulose-xyloglucan composites showed a transition from ordered to disordered cellulose forms. This was reflected by a decrease in the peak intensity at 1111 cm^−1^ (C-O stretching) and an increase at 895 cm^−1^ (C1 frequency), indicating that pectin and hemicelluloses disrupted hydrogen bonding among the CMF [111]. FTIR spectroscopy has also been used to investigate the interactions between components in the LbL shell of CNC-polyethyleneimine microcapsules, as shown in Fig. 8b. A peak at 3342 cm^−1^, which was associated with the stretching vibrations of OH groups, suggested a shift to a lower wavenumber compared with the expected position at ~ 3500 cm^−1^. Additionally, there was a noticeable shoulder peak at ~ 3290 cm^−1^. These spectral features indicated the formation of hydrogen bonds, suggesting the establishment of hydrogen-bonded networks between CNC and polyethyleneimine [140].

FTIR microspectroscopy is an advanced version of conventional FTIR spectroscopy that uses a microscope to focus the IR beam onto a very small sample area, typically in the range of micrometers [141]. Combining a polarizer with FTIR, referred to as polarized FTIR microspectroscopy, has been used to investigate in situ changes occurring in intact CWs. This technique may provide insights into macromolecular orientation and interactions within the CW networks during deformations [142]. Figures 8c-e present the IR microscope spectra of hydrated onion tissue in original, stressed, and relaxed states, respectively. The spectra were obtained using incident polarized light, aligned both parallel and perpendicular to the stretching direction. Subtraction spectra were then obtained by subtracting the perpendicular polarized spectrum from the parallel one. Variation in the intensity of peaks was used to assess the orientation of CW polysaccharides. In the case of slight directional alignment (Fig. 8c), the parallel (solid line) and perpendicular (dashed line) spectra of onion cells exhibited minimal differences, which was also reflected in the subtraction spectrum. Upon applying a 10-g load (Fig. 8d), positive peaks became stronger in cellulose and polygalacturonic acid vibrational regions (~ 1200–950 cm^−1^). In the subtraction spectrum, new negative peaks were found at ~ 1745 and ~ 1605 cm^−1^, corresponding to the pectin ester and carboxylate groups, respectively. In addition, overlapping the peaks of glycosidic bonds in non-cellulosic polysaccharides (~ 1150 cm^−1^) with the intense cellulose peak (~ 1162 cm^−1^) suggested the co-alignment of polysaccharides with CMF. The difference in the intensity of parallel and perpendicular peaks indicated that polysaccharides were oriented in the stress directions. After removing the load (Fig. 8e), the absorbance intensity of peaks underwent a minor decrease compared with that of stressed cells, suggesting a partial recovery of stressed sample and a small elastic deformation. Comparing the peak intensities of the subtraction spectra between the original and stressed tissues further suggested a significant plastic deformation of the CW, indicating how mechanical stress affected the structural alignment and molecular interactions within the wall [143].

Polarized FTIR microspectroscopy has also been used to investigate the orientation of particular functional groups in the cell elongation directions [141, 143]. The double-bladed apertures of the microscope were used to set the focal range for a specific area of CW, and spectra from that area were recorded by aligning the polarizers both parallel and perpendicular to the long axis of cell. The difference between the spectra of elongated carrot CWs with polarizers oriented parallel and perpendicular showed the transversal orientation of vibrational modes of esters (~ 1740 cm^−1^), amides (~ 1650 and 1550 cm^−1^), and phenols (~ 1490 and 1600 cm^−1^). This confirmed that the bonds within the carbohydrate region were aligned with the long axis of elongated cells [141, 142].

#### Nano-FTIR Spectroscopy

Nano-FTIR is an advanced technique that integrates IR spectroscopy with AFM-enabled high-resolution spatial scanning, yielding the nanoscale chemical bond mapping of materials at ultrasmall quantities that would otherwise be nontrivial to achieve via conventional spectroscopy methods [144, 145]. Uncovering the spatial organization of biopolymers is integral to the formation and properties of CW-like materials. In nano-FTIR, the AFM tip, acting as an optical antenna, in the focused IR radiation region creates an enhanced near field at the tip apex, resulting in a spatial resolution in the order of tip radius. The near fields penetrate into the sample, enabling probing the subsurface material. The technique surpasses the Abbe’s diffraction limit (5–10 µm for conventional IR), enabling a resolution of 10–20 nm [145]. In a study on the CWs of intact and mutant poplar trees, nano-FTIR spectra of the SCWs and MLs of three poplar species (low lignin, high lignin, and low recalcitrance mutant) yielded the spatial variation in the composition and quantity of biopolymer building blocks. Figure 8f presents nano-FTIR spectra of SCW and ML of poplar fibers along with the detected scattering intensity versus the location of AFM tip for wavenumbers pertaining to polysaccharides and lignin. Polysaccharides and lignin were distinguished via the peaks at 1162 and 1269 cm^–1^, respectively. Nano-FTIR absorbance versus tip location on the CW uncovered that cellulose in the ML of poplars had a disordered structure with a greater spatial variability in polysaccharide and lignin contents compared with the SCW [124]. This technique may be valuable for analyzing CW-like materials, particularly those fabricated via LbL assembly.

#### SFG Spectroscopy

SFG spectroscopy is a non-linear optical technique that probes molecular vibrations at surfaces and interfaces, with inherent sensitivity to non-centrosymmetric structures within an optical medium. [146]. In SFG, the tunable IR laser pulses are combined with the up-conversion laser pulses (i.e., near-IR or visible) at the sample surface, resulting in the emission of new photons at the frequency that corresponds to the sum of the two input photons (ω_SFG_ = ω_visible_ + ω_IR_, ω is the angular frequency of laser pulses, and subscripts visible and IR denote visible light and IR, respectively). When a sample absorbs ω_IR_, the SFG yield increases, and the resulting SFG spectrum contains vibrational spectral features. SFG is functional only for non-centrosymmetric materials. Therefore, the centrosymmetric amorphous medium is SFG-inactive [128]. In plant CWs, only crystalline cellulose, adopting the P2_1_ space groups, fulfills the non-centrosymmetric requirement for SFG measurements. The P2_1_ space group represents a monoclinic crystal system for crystalline cellulose with twofold rotational symmetry along one axis. Other amorphous polysaccharides in the plant CW do not produce SFG signals. Hence, SFG selectively identifies crystalline cellulose segments interspersed in amorphous matrices, enabling the investigation of crystalline properties of native cellulose in PCW and lignocellulosic biomass in their native form without requiring the separation of building blocks [128, 147].

SFG spectroscopy has been used to study the mesoscale polarity (unidirectional versus bidirectional) of CMF in intact plant CWs [128, 148–151]. One study used SFG spectroscopy to investigate the 3D CMF assembly in various PCWs, tunicate, and bacterial pellicles at the mesoscale (i.e., between nm and μm). The features of SFG spectra, such as the overall intensity and shape of peaks, were associated with the CMF assembly in each native material. As shown in Fig. 8g, studies on randomly packed and uniaxially aligned CNC films demonstrated that when cellulose was aligned through stretching, the SFG spectrum contained a strong signal at 2944 cm⁻^1^ (corresponding to the alkyl stretch region) and a weak signal at 3320 cm⁻^1^ (in the hydroxyl stretch region) [148]. This effect arises from the antiparallel ordering of nanocrystals during alignment, where the number of crystals oriented in one direction nearly equals those oriented in the opposite direction. This symmetry cancellation between the OH dipoles of antiparallel CMF results in a minimal net polarity of hydroxyl groups. Disturbing the interactions between the nanocrystals resulted in randomly packed CNC films, the introduction of peaks at 2920 and 2968 cm^−1^, and an increase in the intensity of hydroxyl peak [148]. To study the CMF assembly in various intact CWs, the characteristic peaks of SFG spectra were compared. In the SCW of land plants including flax, ramie, and cotton, and the vascular tissues of poplar, pine, *Arabidopsis*, *Brachypodium*, switchgrass, and maize, the SFG spectra contained a single major peak at 2944 cm^−1^ and small peaks in the hydroxyl stretching region (Fig. 8h), closely resembling the features of the film with antiparallel aligned CNC. However, the spectra of bacterial biofilms, algal CWs, tunicate cellulose samples, and PCWs contained multiple peaks at 2920, 2944, and 2968 cm^−1^ as well as larger hydroxyl peaks (Fig. 8i), resembling the spectra for films with non-aligned CNC [148]. Table 2 presents a summary of advantages and disadvantages of various morphological/structural, mechanical, and chemical techniques used for the characterization of artificial plant CW-like materials.

**Table 2:** Advantages and disadvantages of CW-like material characterization methods

	Technique	Advantages	Disadvantages
Morphological/Structural	AFM imaging	High-resolution nanoscale mapping of CW topography and structure [92–94] Minimal sample preparation, preserving hydrated states and near-native conditions [27] Visualization of individual fibers and structures, such as xyloglucan assemblies [95] Measuring both topography and mechanical properties (e.g., stiffness) [96]	Limited to surface measurements, rendering it difficult to probe internal structures [92] Time-consuming scanning processes, especially for large areas [27] Requiring careful calibration for accurate dimension measurements in hydrated samples [95]
	SEM/TEM imaging	Revealing the internal structures and alignment of CMF and other CW biopolymers at the nanoscale [97, 98] Preserving sample morphology via deep-etch freeze-fracture TEM and preventing network disruption by ice crystals [46] Detecting thin strands and bridging structures between fibers, such as xyloglucan linking CMF [96]	Requiring thin sample preparation (TEM), which may lead to sample distortion or artifacts [46] Time-intensive preparation and imaging processes [97] Limited to small sample areas, rendering large-scale analyses difficult [46] Analyzed in dehydrated states
	QCM	Surface-sensitive, real-time measurement of mass changes during adsorption processes [64, 99, 100] Requiring minimal sample preparation [65] Monitoring layer thickness in CW-mimetic materials [65]	Typically limited to thin, rigid layers for accurate mass calculation via the Sauerbrey equation [99] May not account for viscoelastic properties of softer layers [65]
	QCM-D	Monitoring mass changes and viscoelastic properties of adsorbed layers [54, 101] Uncovering complex interactions between polysaccharides and other CW components [54] Enabling adsorption kinetics studies in real-time, providing insights into the dynamics of CW component interactions [40, 87] Applicable to varying interfaces, including emulsions and films [87]	Complex data interpretation of dual parameters (Δ*f* and Δ*D*) [54] Requiring careful calibration and setup, rendering it time-consuming [54] Sensitivity to environmental conditions, which may affect accuracy [54] Limited to specific materials that adhere well to the sensor surface [54]
	SAXS	Providing information on the size, orientation, and arrangement of CW components without requiring extensive sample preparation [103, 104] Effective for studying hydration effects and structural changes in plant fibers [108] Uncovering CMF orientation in both native and synthetic CWs [109]	Limited to nanoscale features, which may not provide a complete picture of larger structures [103]
	WAXS	Determining crystallinity, crystal type, and cellulose alignment within CW-like composites [103, 111] Analyzing the effects of processing conditions (e.g., wet-spinning) on fiber orientation [112] Revealing changes in cellulose structure under stress, providing insights into load-bearing properties [115]	Requiring crystalline samples, rendering it less effective for amorphous materials [103] Diffraction peaks of crystalline phases are very broad due to the low scattering cross-sections of C and O (zero for H) and small crystal sizes Challenging sample preparation in hydrated states
	SANS	Uncovering hydrogen bonding patterns and complex structural arrangements in CW materials [117, 119] Utilizing deuterated components to enhance the visibility of specific interactions [118] Providing insights into solvent accessibility and interactions between polysaccharides and cellulose [119]	High operational costs [152] Lower flux of neutron sources than X-ray sources, limiting their use in studies involving rapid, time-dependent processes [152] Requiring large sample quantities for accurate measurements [152]
Mechanical	Uniaxial mechanical testing	Providing quantitative measures of mechanical properties, including tensile strength, Young’s modulus, and the work of fracture [10, 51, 53] Mimicking in planta phenomena, such as turgor pressure and creep [39, 51, 121]	Non-trivial measurement of CW component contributions to overall mechanics as a result of plant tissues heterogeneity [121] Inconsistency in creep behavior data as a result of enzymatic activities [121]
	Biaxial mechanical testing	Mimicking real-life biaxial deformations, experienced by plant CWs during expansion [51] Enabling uniform cell stretching and pressure–displacement relationships investigations [51] Suitable for highly hydrated CW-like composites [51]	Requiring specialized equipment, which may limit accessibility and increase testing complexity [51] Challenging analyses under dynamic conditions [51]
	AFM nano-indentation	High-resolution force-distance measurements at small scales, providing detailed mechanical properties, such as elastic modulus and hardness [69, 86] Suitable for analyzing microscopic layers within CWs and CW-like materials [122, 123] Imaging structural aspects like MFA in SCWs [130] Applicable to both soft PCWs and stiffer SCWs with model selection tailored to material properties [127] Analyzing out-of-plane indentation mechanics for soft, thin PCW samples [49]	Requiring appropriate contact models (e.g., Hertz, JKR, DMT) to calculate the modulus [69, 124] Affected by factors such as turgor pressure and complex geometries [133] Certain models (Hertz) lacking viscoelastic contributions, which fail in biological sample analyses [125, 126] Unsuitable for the in-plane compression measurements of soft materials [49]
Chemical	NMR spectroscopy	Providing detailed molecular insights into the local chemical environment of specific nuclei, such as ^13^C and ^1^H [134, 135] Quantifying cellulose crystallinity in CWs and CW-like composites [47] Isotopic labeling (e.g., ^13^C) for site-specific hydration and polymer interactions in native plant tissues [37]	Time-consuming and requiring significant sample preparation, especially for isotopic labeling [37] Inconsistency between crystallinity values from NMR and other techniques due to varying detection principles [136]
	SS-NMR spectroscopy	Studying solid samples, including CWs in both hydrated and dry states [37] Providing high-resolution data on interactions between cellulose, hemicellulose, and lignin [47, 120] Uncovering site-specific hydration effects on CWs [139] Revealing spatial proximities and intermolecular cross-peaks, providing insight into lignin-polysaccharide interactions [137, 138]	Limited sensitivity to dynamic interactions compared with liquid-state NMR [134] Complex data interpretation, especially with overlapping signals from similar components [136] Averaged information of a large amount of samples, consisting of different types of plant tissues (because in many cases they cannot be separated physically)
	FTIR spectroscopy	Providing detailed information on chemical functionality, molecular conformation, crystallinity, and hydrogen bonding [9, 128, 129] Probing interactions between CW components in biologically relevant environments [68, 70, 111, 140] Detecting cellulose allomorph shifts and hydrogen bonding disruptions [111]	Limited spatial resolution, which may not capture fine details in heterogeneous samples [141] Complicated data interpretation as a result of overlapping peaks in complex mixtures [142]
	FTIR microspectroscopy	High spatial resolution (micrometer scale), enabling the localized analysis of CW structure and dynamics [141] Provides insights into macromolecular orientation and interactions under stress via polarized FTIR microspectroscopy [142] Enabling the in situ analysis of CW deformation and recovery in real-time [143]	Requiring more sophisticated equipment compared with conventional FTIR [141] Requiring advanced analysis tools for data interpretation due to complex vibrational modes [143]
	Nano-FTIR spectroscopy	Combining IR spectroscopy with AFM for the nanoscale chemical mapping of materials [144, 145] Achieving high spatial resolution (~ 10–20 nm), surpassing the diffraction limit of conventional IR (~ 5–10 µm) [145] Providing detailed chemical composition and spatial variability of biopolymers, enabling subsurface analyses [124]	Challenging data interpretation, especially for heterogeneous materials, such as CWs [145] Limited accessibility due to the specialized nature and technical expertise required for operation [124] The realistically achievable resolution is lower than the best case demonstrated by the vendor using control samples
	SFG spectroscopy	Resolving crystalline structures within amorphous matrices [128, 147] Sensitive to molecular vibrations at surfaces and interfaces, ideal for studying CW component interactions [146] Non-invasive, enabling sample analyses in their native, hydrated states [128, 147]	Limited to non-centrosymmetric materials, excluding amorphous polysaccharides and other CW components [128] Requiring specialized equipment and expertise, limiting its accessibility [146] Complex data interpretation, especially when analyzing complex biological materials, such as CWs [148] Theory is still in the developmental stage [153]

## Applications of CW-Like Materials

CW-like materials have been developed to uncover the complex structure–property relationships of natural CW components or to fabricate functional soft materials for real-life applications. These materials are relevant to a wide variety of research fields and industries, such as biology, materials science, renewable energy, pulp and paper, agriculture, biomedicine, and food science. In the development of CW-like materials, plant CW constituents such as cellulose, hemicellulose, pectin, and lignin are frequently used. These materials are valued for their widespread availability, low cost, biodegradability, renewability, and non-toxicity [38, 154, 155]. Here, we provide examples of the current and potential applications of plant CW-mimetic materials.

Studying the mechanical and rheological properties of synthetic plant CWs may uncover interesting information about the textural properties of native vegetables and fruits [53]. CW models composed of bacterial cellulose, pectin, and xyloglucan have been studied alongside native apple tissues in the presence of calcium, which plays a crucial role in maintaining the texture and firmness of fresh fruits. It was found that the calcium impact on the mechanical properties of artificial CW closely mirrored its effect on the native apple tissue. Additionally, higher pectin and xyloglucan contents were associated with a softer and less crispy fruit texture, resulting in greater extensibility. In this regard, synthetic CW composites made up of intact cellulose matrices from the corresponding native materials offer more accurate models for mimicking natural CW mechanics than reconstituted plant tissue films, especially at high relative humidity. Water sorption is a critical factor in tuning food texture. When fruits are stored, water loss through the skin can cause them to shrivel and lose quality. To this end, synthetic CWs were used as models to investigate the effects of water and humidity on food texture and to explore their potential as texture modifiers for fruits and vegetables [40]. The synthetic CW composites, which included bacterial cellulose, pectin, and xyloglucan, underwent reduced water retention, water conductivity, and diffusivity compared with cellulose alone. This decrease was attributed to the reduction in void spaces as additional components were incorporated. The values obtained were comparable to those of real plant tissues, underscoring the suitability of this composite for mimicking the native tissue.

Food wastes resulting from spoilage and improper packaging remain a challenge in developing a more sustainable society [156]. Pectin and cellulose films that mimic the mechanical and barrier properties of plant CWs have emerged as a solution to address this issue. Edible pectin films cater to the consumer demand for minimally processed foods and can be imparted with antimicrobial materials, such as silver ions, to extend shelf life and reduce pathogen growth [157, 158]; however, polysaccharide-based films are known to be susceptible to moisture sorption. The film composition significantly influences its moisture sorption properties [159, 160]. The addition of lignin may improve the moisture resistance of films, as demonstrated by several studies [74, 161, 162]. For example, a composite film made from CNF with 25% lignocellulose CNF (containing 16% preserved lignin) yielded a 16% decrease in water vapor transmission rate (WVTR) compared with a neat CNF film. This decrease was attributed to lignin, performing as a chemical adhesive among the CNF, which impeded gas transmission through the film [162].

Biomass microcapsules with a CW-like shell offer tunable permeability, depending on the shell composition and fabrication method. These microcapsules have been used to deliver therapeutics through the gastrointestinal (GI) tract by leveraging factors such as pH and salinity, or to deliver cells to tissues via the porous shells. Several studies have reported promising results with CW-like microcapsules for these applications [83, 140, 163, 164]. For instance, the stimuli-responsive microcapsules containing pectin, xyloglucan, and CNF showed reduced permeability to FITC-dextran molecules in a saline solution (or biological media) due to the NaCl-mediate screening of charged polymers. When rinsed with water, the FITC-dextran molecules were subsequently released. The biocompatible nature of microcapsules may provide opportunities for targeted release of biologics in the GI tract [83].

Engineering CW-like materials for construction applications is crucial for a bio-based economy and addressing the demand for carbon-negative building materials. These materials may actively contribute to reducing carbon dioxide production [165, 166]. Lumber and engineered wood are well-established materials in construction and manufacturing due to their low density and sustainability, particularly when compared with steel or concrete. However, the slow growth cycle of timber-grade trees limits the widespread use of lumber-based materials [167, 168]. Wood-like composites prepared from biomass have been developed as a class of tough, hydrophobic biopolymer composites, which may supplement the timber industry [71, 73, 169]. Artificial lignification has enabled the fabrication of CW-like materials with hydrophobic characteristics, resembling natural analogs. Note that the materials developed thus far have not replicated the anisotropic properties of wood, and to the best of our knowledge, no study has demonstrated a bottom-up approach by which lignified materials with a high degree of anisotropy are fabricated. Many studies have developed bottom-up approaches to produce highly aligned CMF within cellulose-only composites [112, 114, 170]. These materials have high strength and stiffness, but are often brittle and water sensitive [171, 172]. Studies on artificial CW have shown that incorporating low to moderate amounts of hemicellulose or lignin fills the interstitial voids among CMF, which improves stress transfer and increases composite ductility and toughness [74, 77]. Recapitulating cellulose networks, in which long CMF are aligned to promote extensive inter-fibril interaction, with matrix polymers serving as fillers to enhance this interaction, is essential for replicating the strength and extensibility of native CW.

In the pulp and paper industry, separation of cellulose from lignin and hemicellulose via thermomechanical and chemithermomechanical pulping are resource intensive. These processes require significant amounts of energy, raw materials, time, and labor; however, they may be optimized via understanding the interactions of cellulose, hemicellulose, pectin, and lignin within plant PCW and SCW. Through such explorations, a low degree of sulfonation pretreatment was applied to spruce wood chips, selectively modifying the PCW without causing any ultrastructural damage to the cell architecture. Sulfonation not only weakened the interactions between lignin-pectin, lignin-protein, and pectin–protein, but also softened and swelled the stiff, elastic materials. This was ascribed to the weakening of lignin-pectin bonds specifically, which loosened the pectin network. As a result, the increased softness of materials enabled significant electrical energy saving (up to 200 kWh t^−1^) during the refining process of chips [173, 174].

Understanding the interactions among CW components may help convert biopolymers to chemical feedstocks. Synthetic CWs have been fabricated to identify the kinetics and thermodynamics of biopolymer adsorption to cellulose, along with the effects of matrix polymers on cellulose crystallization and lignification within cellulose networks [54, 90, 175]. These investigations may help increase the economic value of biomass by diversifying the range of chemical feedstocks derived from native CW components. As an example, lignin content or composition has been genetically engineered to facilitate the deconstruction of plant CW. This is in line with the “lignin-first” biorefinery concept, which entails isolating lignin catalytically without compromising carbohydrates, such as cellulose and xylan, to produce valuable aromatic derivatives. This concept is integral to the efficient recovery of carbon from biomass due to the recalcitrance and high content of lignin (e.g., ~ 25%) [176]. The intricate structure of plant CW components and their complex interactions, which may be better understood through plant-mimetic CW systems, hold significant potential for the production of valuable target materials, such as thermosets, thermoplastics, composites, CNC, and nanofibers [177–179].

Recently, there has been a growing interest in utilizing CW-mimetic materials to synthesize specialty chemicals via biochemical processes. In the biocatalysis sector, artificial CWs have been developed that contain catalysts to facilitate cascade reactions [180]. As an example, artificial plant CW-like materials containing cellulose and other polysaccharides were doped with lipase and palladium nanoparticles. In organic solvents (toluene, 1,4-dioxane, or 3-methyl-3-pentanol), the composites converted racemic amine (equal amounts of two enantiomers) to an enantiomerically pure amine ((R)-2-methoxy-N-(1-phenylethyl)acetamide) with high yields (~ 70%-81%) [180]. The CW-mimetic materials facilitated efficient enzyme activation in organic solvents by immobilizing enzyme on cellulose, which served as the support material. This approach allowed for multiple cycles of enzyme reuse, significantly lowering the cost of chemical production. Altogether, CW-mimetic materials have been used in antimicrobial food packaging, edible films, stimuli-responsive microcapsules, lubricants for stress transfer, carbon-negative building materials, and heterogeneous biocatalysis. Thus, beyond providing a fundamental platform for understanding native plant CWs, CW-mimetic soft materials may open new opportunities in circular economy and sustainability.

## Conclusions

Plants have remarkable mechanical and structural properties originating from hierarchical CWs, complex interactions among CW components, and their precise alignment and assembly. There are limitations in studying the properties of CW components and layers directly in planta, leaving key questions unanswered about how plants construct their CWs. Bottom-up assembly approaches using individual CW components may provide valuable insights into the mechanical properties, spatial organization, and biopolymer interactions in plant CWs, aspects that are often difficult to investigate directly within plants. Moreover, CW-like materials fabricated via bottom-up approaches hold promise across diverse fields, such as pulp and paper, specialty chemicals, biomedicine, food science, and packaging. The fabrication methods include bacterial growth and impregnation, LbL assembly, film casting, 3D templating of microcapsules, and particle coating, all of which can be guided by in silico modeling. Although these techniques have successfully enabled the development of artificial CWs that mimic the structural and functional properties of natural CWs, scaling them up for industrial applications remains a significant challenge. For example, bacterial cellulose growth and LbL assembly are time-intensive processes, which may limit their economic viability for large-scale production. While film casting and particle coating methods are more straightforward, they often require highly controlled conditions, such as specific humidity, temperature, pH, and solvents, which may be challenging to maintain in an industrial setting. Additionally, the integration of these methods into existing manufacturing processes requires significant modifications in terms of equipment and processing protocols. To advance the applicability of artificial plant CWs in industrial contexts, future research may focus on developing scalable fabrication techniques. Addressing these challenges may require optimizing existing methods to reduce processing time and cost or developing entirely new approaches that are inherently scalable and suited for mass production. For example, combining additive manufacturing technologies with advanced biomaterials may offer a promising pathway for the rapid and cost-effective production of CW-like materials at scale. Collaboration between academia and industry will be essential to successfully translate these laboratory-scale methods into practical, commercially viable solutions.

Natural plant CWs and CW-mimetic materials require detailed mechanical, chemical, morphological, and structural characterizations to uncover their intricate properties and unlock their full potential for various applications. For the mechanical analysis of CWs, multiaxial mechanical tests, AFM nano-indentation, and nano-FTIR spectroscopy have been carried out. Additionally, 1D/2D NMR, FTIR, and SFG spectroscopy have provided fundamental information on the interactions of CW components in native, hydrated, and stressed states. Morphology, crystalline structure, and intermolecular/interfibrillar orientations/interactions have been analyzed using microscopy, including AFM, TEM, and SEM, as well as scattering techniques (e.g., XRD, SAXS, WAXS, and SANS) and QCM-D, respectively. Efforts to replicate natural CWs have led to new insights into the structure–property relationships of CW biopolymers. While CW-mimetic materials provide valuable information on the interactions among plant CW components, the accuracy and applicability of these insights to real CW warrants further validation. Current CW-mimetic materials still lack the ability to replicate key CW properties, such as the cross-lamellate wall structure of PCWs, integration of lignin in the SCWs, and stress–strain behaviors. Notably, mimicking CWs may enable the construction of strong, lightweight, and sustainable composites, biocompatible carriers for medical applications, and biodegradable and smart packaging, among many other applications contributing to the United Nations sustainable development goals. Overall, via reviewing current literature on CW-mimetic materials, this paper aims to serve as a resource for guiding the fabrication, characterization, and application of new generations of plant CW-mimetic soft materials.
